# Supplementary material for: Enhanced restoration of visual code after targeting ON bipolar cells compared with retinal ganglion cells with optogenetic therapy
Source: Mol Ther. 2025 Jan 17;33(3):1264–81. doi: 10.1016/j.ymthe.2025.01.030 (PMC11897768; doi:10.1016/j.ymthe.2025.01.030)
Supplement: Document S1. Figures S1–S4 [file mmc1.pdf]

YMTHE, Volume *33*

Supplemental Information

Enhanced restoration of visual code after targeting ON bipolar cells compared with retinal ganglion cells with optogenetic therapy

Jessica Rodgers, Steven Hughes, Aghileh S. Ebrahimi, Annette E. Allen, Riccardo Storchi, Moritz Lindner, Stuart N. Peirson, Tudor C. Badea, Mark W. Hankins, and Robert J. Lucas

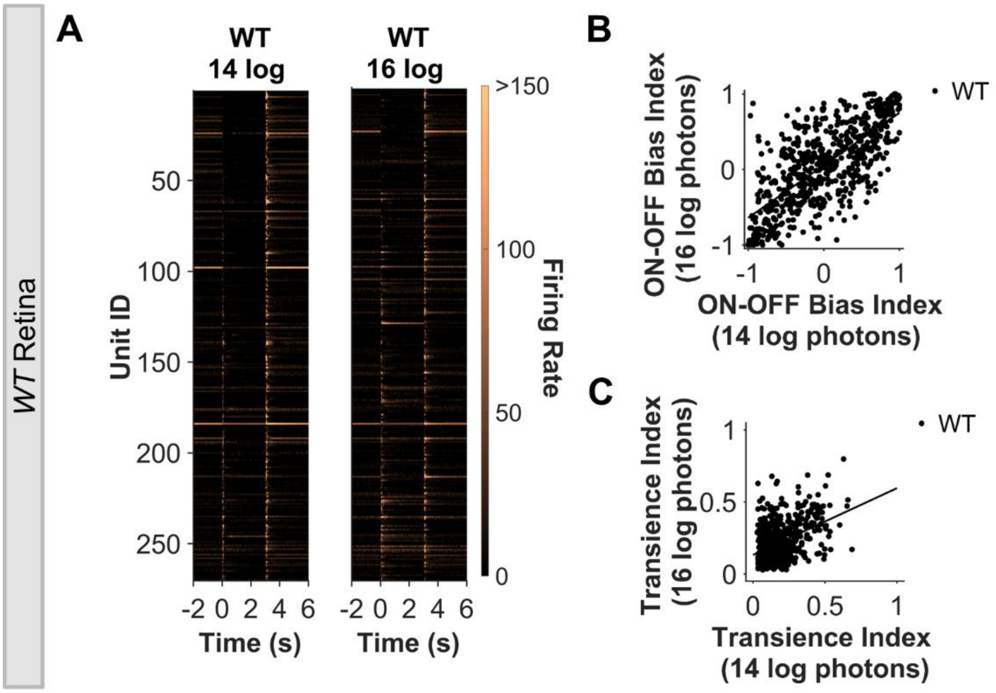

Figure S1

A) Heat map of mean PSTH for step stimulus (ON from 0-3s) for LR units in WT retina ordered by bias index from OFF (top) to ON (high) under 13.95 and 15.95 log photons/cm^2/s. Each row represents an individual unit. In the left and right heatmaps, each row represents the same unit recorded under different conditions (ranked based on ON-OFF bias for 13.95 log photons).

B) Scatterplot of ON-OFF bias index for same WT units at 13.95 and 15.95 log photons/cm^2/s.

C) Scatterplot of Transience index for WT units at 13.95 and 15.95 log photons/cm^2/s.

Data are from N = 630 WT retinal units (must be LR under both intensities to be included)

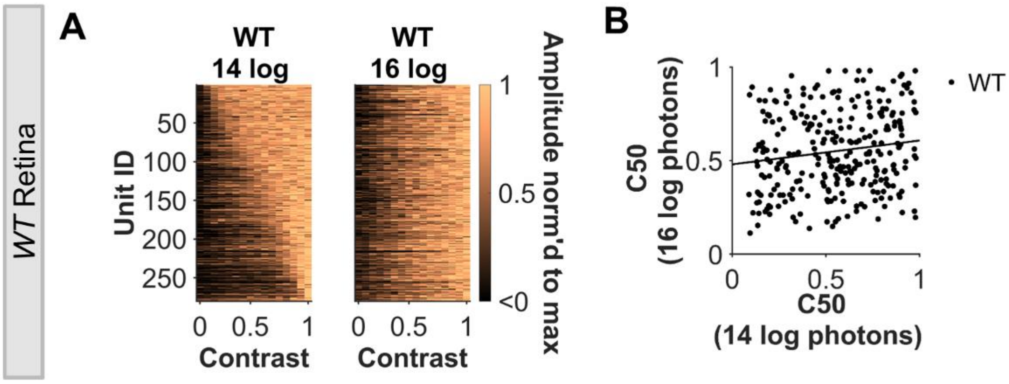

Figure S2

A) Heat map of maximum normalised response amplitude across contrasts for LR WT retinal units. Each row represents an individual unit. In the left and right heatmaps, each row represents the same unit recorded under different conditions (ranked by C50 for 13.95 log).

B) Scatterplot of C50 for same WT units at 13.95 and 15.95 log photons/cm^2/s.

Data are from N = 280 WT retinal units.

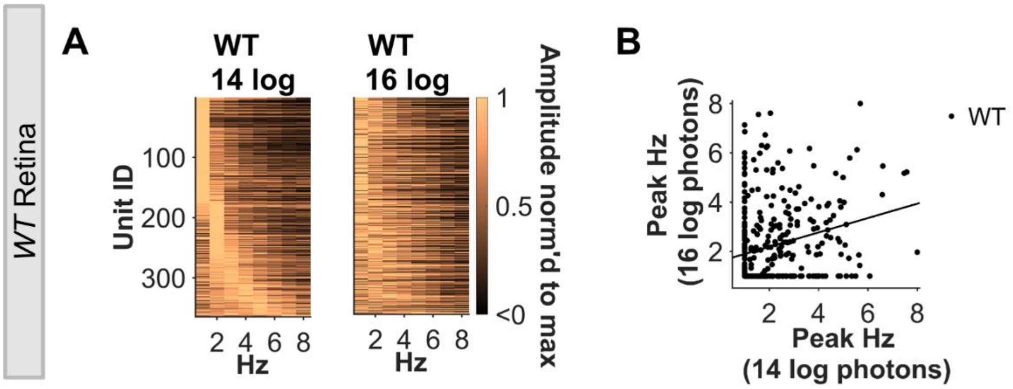

Figure S3

A) Heat map of maximum normalised response amplitude across frequencies for LR WT retinal units. Each row represents an individual unit. In left and right heatmaps, each row represents the same unit recorded under different conditions (ranked by peak TF for 13.95 log photons). **B)** Scatterplot of peak temporal frequency (peak Hz) for same WT units at 13.95 and 15.95 log photons/cm^2/s.

Data are from N = 369 WT retinal units.

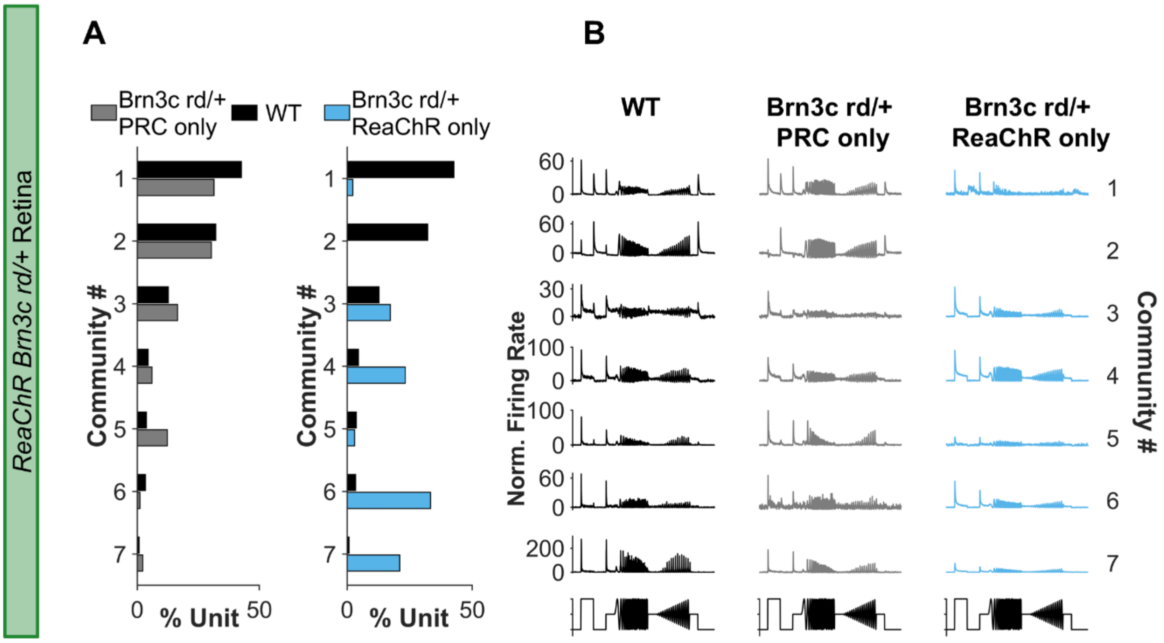

Figure S4

Community detection used data from LR retinal units at 13.95 photons/cm^2/s (WT and Brn3c rd/+ PRC only) and 15.95 photons/cm^2/s (Brn3c rd/+ ReaChR only). Units were randomly down-sampled to match sample size of genotype with fewest units, n = 267 units for each group. After discarding sPCs accounting for <1% of variance, we extracted 52 sPCs. Data for Brn3c rd/+ PRC and ReaChR only groups is within-subjects (ie: units are identical for both conditions), recorded under different conditions (see Figure 4b for further details). Data from each genotype is shown for communities with sufficient units to calculate mean (N ≥ 3).

A) Distribution of units across communities.

B) Mean baseline-subtracted firing rate for units from each community
