# Supplementary material for: Enhanced restoration of visual code after targeting ON bipolar cells compared with retinal ganglion cells with optogenetic therapy
Source: Mol Ther. 2025 Jan 17;33(3):1264–81. doi: 10.1016/j.ymthe.2025.01.030 (PMC11897768; doi:10.1016/j.ymthe.2025.01.030)
Supplement: Document S2. Article plus supplemental information [file mmc2.pdf]

Molecular Therapy
Original Article

# Enhanced restoration of visual code after targeting ON bipolar cells compared with retinal ganglion cells with optogenetic therapy

Jessica Rodgers,[1,8] Steven Hughes,[2,3,8] Aghileh S. Ebrahimi,[1] Annette E. Allen,[1] Riccardo Storchi,[1] Moritz Lindner,[2,4,5] Stuart N. Peirson,[2,3] Tudor C. Badea,[6,7] Mark W. Hankins,[2,3] and Robert J. Lucas[1]

[1]Faculty of Biology, Medicine & Health, University of Manchester, Manchester M13 9PT, UK; [2]Nuffield Laboratory of Ophthalmology, Sleep and Circadian Neuroscience Institute, Nuffield Department of Clinical Neurosciences, University of Oxford, Oxford OX1 3QU, UK; [3]Kavli Institute for Nanoscience Discovery, University of Oxford, Oxford OX1 3QU, UK; [4]Institute of Physiology and Pathophysiology, Department of Neurophysiology, Philipps University, 35037 Marburg, Germany; [5]Department of Ophthalmology, University Hospitals of Giessen and Marburg, 35043 Marburg, Germany; [6]Neurogenetics Laboratory/ICDT, Transilvania University of Brasov, 500484 Brasov, Romania; [7]National Brain Research Centre/ICIA, Romanian Academy, 050711 Bucharest, Romania

**Optogenetic therapy is a promising vision restoration method where light-sensitive opsins are introduced to the surviving inner retina following photoreceptor degeneration. The cell type targeted for opsin expression will likely influence the quality of restored vision. However, a like-for-like preclinical comparison of visual responses evoked following equivalent opsin expression in the two major targets, ON bipolar (ON BCs) or retinal ganglion cells (RGCs), is absent. We address this deficit by comparing stimulus-response characteristics at single-unit resolution in the retina and dorsal lateral geniculate nucleus of retinally degenerate mice genetically engineered to express the opsin ReaChR in *Grm6*- or *Brn3c*-expressing cells (ON BC vs. RGCs, respectively). For both targeting strategies, we find ReaChR-evoked responses have equivalent sensitivity and can encode contrast across different background irradiances. Compared with ON BCs, targeting RGCs decreased response reproducibility and resulted in more stereotyped responses with reduced diversity in response polarity, contrast sensitivity, and temporal frequency tuning. Recording ReaChR-driven responses in visually intact retinas confirmed that RGC-targeted ReaChR expression disrupts visual feature selectivity of individual RGCs. Our data show that, while both approaches restore visual responses with impressive fidelity, ON BC targeting produces a richer visual code closer to that of wild-type mice.**

## INTRODUCTION

Optogenetic therapy is a promising vision restoration approach for retinal degeneration. In dystrophic retinas with lost rod and cone photoreceptors, the missing visual input can be replaced by genetically engineering surviving retinal cells to express light-sensitive proteins called opsins.[1] This approach is appropriate for patients suffering photoreceptor loss regardless of underlying cause and has been validated in numerous preclinical studies employing animal models of retinal degeneration.[2,3] Several clinical trials are underway, with an early report of successful, albeit limited, vision restoration.[4]

An important decision point for any ocular gene therapy intervention is which cell type(s) in the retina to target for transgene expression. The target population can be clear with gene replacement, such as *LRIT3* therapy for congenital stationary night blindness in ON bipolar cells (ON BCs)[5,6] or *CNGB3* therapy for achromatopsia in cone photoreceptors.[7,8] However, in the case of optogenetic therapy, the ideal cell target is less obvious. The suitability of AII amacrine cells[9] and surviving cones[10] have been explored, but most work on this topic has focused on either ON BCs[11–17] or retinal ganglion cells[18–25] (RGCs). These two cell types offer distinct advantages and limitations. In favor of RGCs is ease of transduction and resilience in the face of progressive degeneration. RGCs are the main population transduced following intravitreal injection using ubiquitous promoters, such as CAG or CMV, with available adeno-associated virus serotypes.[26] In comparison, BCs are more challenging to transduce and require a combination of cell-specific promotors[6,12,27–33] and modified capsids.[34–36] RGCs are also less affected by the retinal reorganization that accompanies progressive degeneration. In comparison, BCs are more subject to cell death and exhibit greater morphological and genetic changes after retinal degeneration than RGCs.[37–39] In principle, this makes RGCs more reliable hosts for optogenetic actuators.

Received 24 July 2024; accepted 14 January 2025;
https://doi.org/10.1016/j.ymthe.2025.01.030.

[8]These authors contributed equally

**Correspondence:** Mark W. Hankins, Nuffield Laboratory of Ophthalmology, Sleep and Circadian Neuroscience Institute, Nuffield Department of Clinical Neurosciences, University of Oxford, Oxford OX1 3QU, UK.
**E-mail:** mark.hankins@eye.ox.ac.uk
**Correspondence:** Robert J. Lucas, Faculty of Biology, Medicine & Health, University of Manchester, Manchester M13 9PT, UK.
**E-mail:** robert.lucas@manchester.ac.uk

 © 2025 The Authors.
Published by Elsevier Inc. on behalf of The American Society of Gene and Cell Therapy.
This is an open access article under the CC BY license (http://creativecommons.org/licenses/by/4.0/).

The major argument in favor of ON BCs is that targeting these cells may better recreate the natural visual code. Here, retinal circuitry upstream of RGCs performs important computations allowing diversity in visual feature selectivity of the retinal output.[40,41] Introducing optogenetic actuators to RGCs runs the risk of replacing this complex visual code with a stereotyped visual response that fails to recreate the expected visual feature selectivity for many ganglion cell types. By contrast, optogenetic signals originating in ON BCs would propagate through inner-retinal circuits allowing the possibility that they will be subject to many of the same computations as native photoreceptor-derived inputs. The most obvious example of such processing is the generation of separate ON and OFF representations of the visual scene and, indeed, ON BC optogenetic interventions can recreate such diversity in the visual code.[12,15,17,42]

Understanding how the visual code is impacted by choosing to target optogenetic actuators to ON BCs vs. RGCs is thus an important step in optimizing this therapy. Previous comparisons of visual response following expression of the same opsin targeted to different cell types have asked whether ON BC targeting strategies offer advantages over approaches biased toward RGC expression.[27,29,43] Based upon population level analyses of light-evoked activity in RGCs these have reported that ON BC targeting can reduce photosensitivity[27,29] (although see Lindner et al.[43]) and alter temporal frequency tuning.[43] However, those studies fall short of a detailed description of the visual code at single-unit resolution as required to reveal its complexity. Moreover, they employed viral gene delivery to achieve opsin expression, introducing unavoidable variability in the amount of opsin expressed across cells and allowing the possibility that any differences in response properties arise from the difference in viral transduction efficiency between cell types.

A clearer picture of the relative advantages of ON BC vs. RGC targeting thus awaits a quantitative comparison of the visual code at single-unit resolution without the variability inherent in viral gene delivery. We have previously used a transgenic mouse line to achieve more uniform and pan-retinal expression of the optogenetic actuator ReaChR across ON BCs and shown that this strategy has an impressive ability to recreate features of the wild-type (WT) visual code when applied to the *Pde6*$^{rd1}$ model of retinal degeneration.[42] Here, we adopted a similar approach to achieve ReaChR expression across a population of RGCs. This facilitated a like-for-like comparison of visual restoration by the same opsin expressed under the same promoter in the two different cell types. We find that both ON BCs and RGCs are suitable targets for high fidelity visual responses, but that targeting ON BCs does indeed better recreate the diversity of the natural visual code.

## RESULTS

### Transgenic mouse models of ON BC- vs. RGC-targeted optogenetic vision restoration

To achieve more standardized opsin expression than is achievable with viral gene delivery, we generated versions of the *Pde6b*$^{rd1}$ mouse model of retinal degeneration[44,45] bearing a floxed ReaChR-mCitrine cassette in the Rosa26 locus,[46] combined with a transgene providing Cre recombinase expression under either *Grm6*[42,47] (termed here ReaChR Grm6 rd/rd) or *Brn3c*[48] (termed here ReaChR Brn3cr rd/rd) promoters. This results in retinally degenerate mice with expression of the red-shifted channelrhodopsin, ReaChR[49] (a light-sensitive ion channel) in either the majority of ON BCs (Grm6 Cre) or in a subset of RGCs[48] (Brn3c Cre). Immunohistochemical analysis of retinal sections from 5-month-old animals confirmed the expected degeneration of the photoreceptor cell layer and revealed ReaChR expression in cells at the outer portion of the inner nuclear layer in Grm6 Cre mice, and in the ganglion cell layer (GCL) in Brn3c Cre mice, consistent with expression in ON BCs and RGCs (Figure 1A). Dendrite stratification from *Brn3c*-expressing RGCs was found in both the on and off sublaminae of the inner plexiform layer, consistent with previous reports that this Cre-driver line targets monostratified ON and OFF, as well as bistratified, RGC subtypes.[48]

### Population-level responses—Amplitude, sensitivity, and sensitivity normalization

We examined responses to a full-field chirp stimulus, previously used to characterize visual response diversity in mice.[40] The chirp comprised a 3 s light step from dark followed by sinusoidal modulations of increasing temporal frequency or contrast (Figure 1B) and was presented at a range of irradiances. To assess visual responses, we first set out to record visually evoked responses in the dorsal lateral geniculate nucleus (dLGN) in urethane-anesthetized mice (Figure 1C). At age 5 months, mice homozygous for the *Pde6b*$^{rd1}$ mutation (termed here rd/rd) have advanced retinal degeneration and dLGN visual responses are restricted to very sluggish and low sensitivity changes in firing driven by the low acuity, inner retinal, photoreceptor melanopsin.[50] As a first assessment of the success of ReaChR expression in either ON BCs or RGCs at restoring visual responses, we reviewed mean firing rate profiles of spike sorted single units from the dLGN of Grm6 or Brn3c rd/rd mice across repeats of the chirp. In both genotypes, high amplitude modulations in mean firing rate associated with the stimulus were apparent at higher irradiances ($n$ = 16 electrode placements from 7 mice for ReaChR Grm6 rd/rd and $n$ = 14 placements from 6 mice for ReaChR Brn3c rd/rd, Figure 1D). Equivalent stimuli did not elicit changes in mean firing rate in rd/rd mice without ReaChR expression. We then quantified response amplitude as the peak change in baseline-subtracted firing rate during or just after the 3 s step across an epoch designed to capture both ON and OFF excitation responses. This parameter was positively correlated with irradiance in both genotypes, with no detectable difference in response to the brightest light (Figure 1E, median = 45 spikes/s for ReaChR Grm6 rd/rd and 47 spikes/s for ReaChR Brn3c rd/rd, U = −0.68, $p$ = 0.493, Mann-Whitney U-test used for statistical comparisons between these genotypes unless otherwise specified). As a more comprehensive assessment of response caliber, we calculated a quality index[40] describing response reproducibility (from 0 to 1, where 1 is identical response to all trials). Quality index was slightly higher in the ON BC compared with RGC-targeted ReaChR, but showed substantial variation across units in both genotypes (median QI = 0.31 for ReaChR Grm6 rd/rd, 0.27 for ReaChR Brn3c rd/rd, U = 2.58, $p$ = 0.009, Figure 1F).

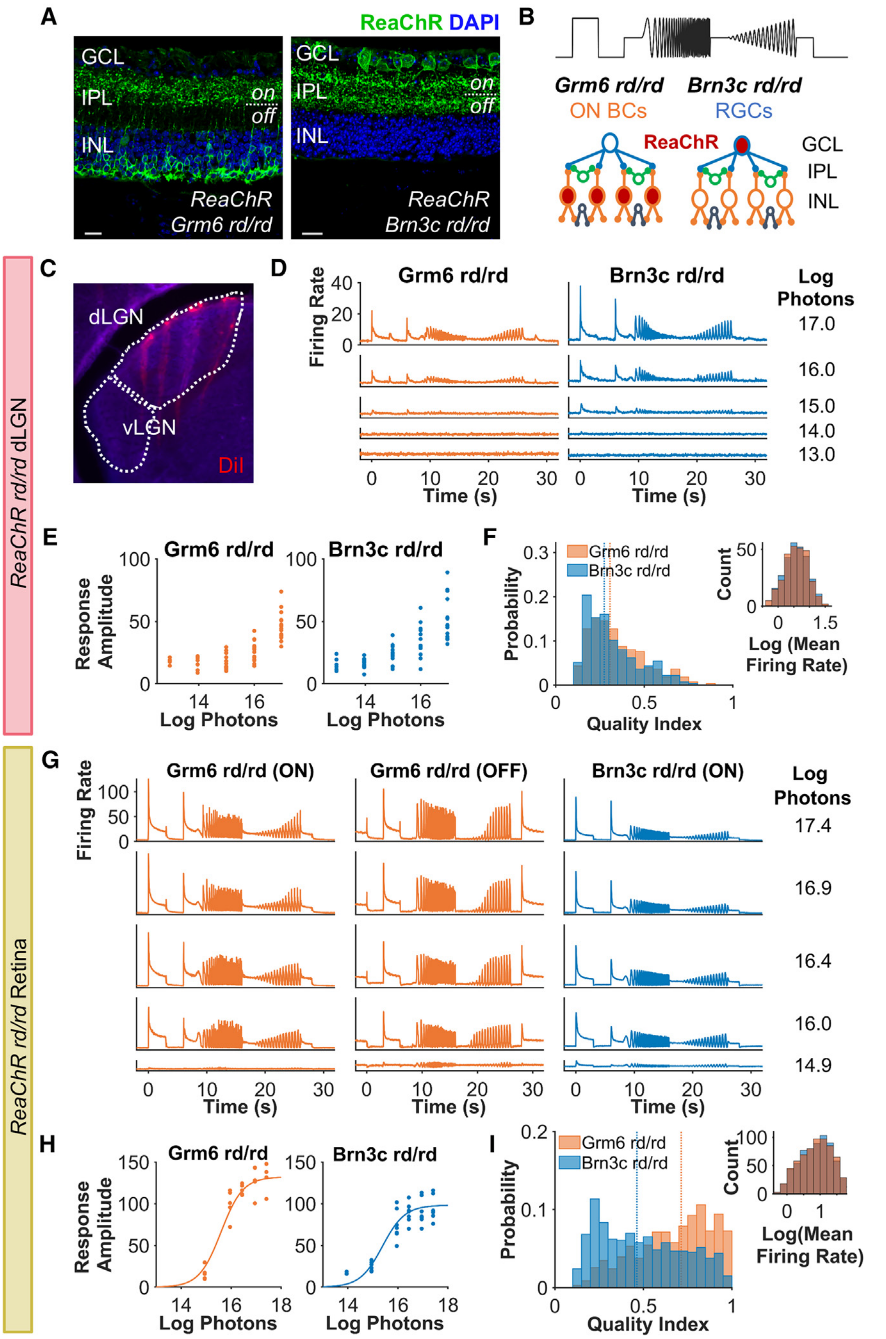


**Figure 1. Population-level responses to light**

(A) Immunohistochemistry from retinal sections from ReaChR Brn3c rd/rd (Brn3c rd/rd) and ReaChR Grm6 rd/rd (Grm6 rd/rd) stained for DAPI (blue) and ReaChR-mCitrine (green). Scale bars, 20 μm. GCL, ganglion cell layer; IPL, inner plexiform layer; INL, inner nuclear layer; on, ON sublamina of IPL; off, OFF sublamina of IPL. (B) Chirp stimulus (top) and schematic of experiment. ReaChR was expressed in a subset of RGCs or ON bipolar cells (ON BCs) and visual responses recorded from RGCs and dLGN neurons using multi-electrode arrays. (C) LGN targeting using multi-electrode arrays. Location of shanks in dLGN during *in vivo* electrophysiology recordings are shown by fluorescent labeling using DiI (red). (D and G) Mean firing rate for light responsive (LR) (D) dLGN units ($n$ = 310 for Grm6 and 348 for Brn3c) and (G) LR retinal units ($n$ = 343 for Grm6 ON, $n$ = 116 for Grm6 OFF, $n$ = 880 for Brn3c) to chirp stimulus across intensities. (E and H) Mean maximum baseline-subtracted firing rate to step stimulus for LR units from (E) each dLGN placement ($n$ = 16 for Grm6 and $n$ = 14 for Brn3c) and (H) each retinal recording ($n$ = 5 for Grm6 and $n$ = 7 for Brn3c) across intensities. Data in (H) are fit with irradiance response curve. (F and I) Histogram of quality index for LR units at brightest intensity tested from (F) dLGN units at 16.97 log photons ($n$ = 275 for both) and (I) retina units at 17.4 log photons ($n$ = 659 for both). Quality index was calculated for a subset of Brn3c and Grm6 rd/rd units matched for average firing rate. Right inset shows distribution of mean firing rate for matched datasets.

Having recorded the thalamic response to visual stimulation, we next turned to a direct assessment of the retinal output by recording extracellular activity across the GCL of retinal explants using multielectrode arrays (MEA). As in the dLGN, the chirp stimulus induced notable modulations in firing rate across the population of GCL neurons, especially at higher irradiances (Figure 1G; plotted separately for units excited vs. inhibited by the light step, termed ON and OFF, respectively, in ReaChR Grm6 rd/rd following Rodgers et al.).[42] Few OFF units were found for ReaChR Brn3c rd/rd, see below, so only ON units are shown for this genotype. To estimate sensitivity, we constructed an irradiance response curve based on change in baseline-subtracted firing rate for each retinal recording ($n$ = 5 retinas from 5 ReaChR Grm6 rd/rd mice and $n$ = 7 retinas from 4 ReaChR Brn3c rd/rd mice). There was no substantial difference in photosensitivity (log $EC_{50}$, intensity that produced half maximum amplitude response, was 15.6 for ReaChR Grm6 rd/rd and 15.4 photons/cm$^2$/s for ReaChR Brn3c rd/rd, Figure 1H), although the response amplitude at brightest intensity tested was attenuated for ReaChR Brn3c rd/rd compared with ReaChR Grm6 rd/rd (105.9 and 138 spikes/s, respectively, U = 2, $p$ = 0.010). Enhanced response amplitude in ReaChR Grm6 rd/rd retinal units was also apparent in the quality index, which was markedly skewed to higher values in this genotype compared with ReaChR Brn3C rd/rd (median QI = 0.71 for Grm6, 0.46 for Brn3c, U = 13.17, $p < 0.001$, Figure 1I).

The vertebrate visual system adjusts its sensitivity according to background light intensity in order to encode visual contrast across large differences in ambient illumination. Although ReaChR-derived

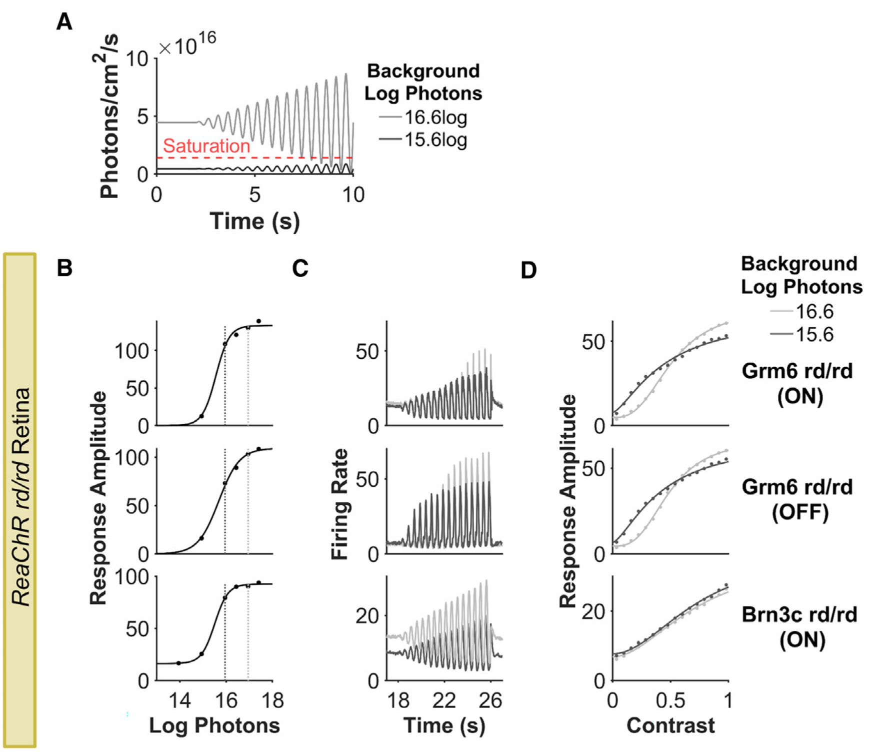


**Figure 2. Sensitivity normalization**

(A) Intensity of contrast chirp stimulus at two background irradiances (16.6 and 15.6 log photons/cm$^2$/s). For responses saturating at 16 log (dashed red line), contrast modulations cannot be tracked without sensitivity normalization. (B–D) Comparison of contrast coding at two different background irradiances, marked by vertical dashed lines, on (B) irradiance response curves; (C) firing rate to contrast chirp stimulus; and (D) contrast sensitivity curves at 15.6 log (dark gray) and 16.6 log (light gray) photons background. Data in (B)–(D) are from $n$ = 343 for ReaChR Grm6 rd/rd ON, $n$ = 116 for ReaChR Grm6 rd/rd Grm6 OFF, $n$ = 880 for ReaChR Brn3c rd/rd retinal units. Units are divided into ON and OFF as described in Rodgers et al.[42] to aid visualization of contrast chirp responses.

vision, irrespective of whether expressed in ON BCs or RGCs, has a high absolute threshold, it is still important to determine if ReaChR-driven responses show sensitivity normalization at higher light levels, as the alternative would be saturation. We therefore compared responses to contrast modulations at two irradiances within the ReaChR sensitivity range. At the higher mean irradiance (light gray line in Figure 2A), most elements of the contrast chirp stimulus lie above the saturating irradiance for responses to the simple light step (red line in Figure 2A). Conversely, all elements of the dimmer chirp (black line in Figure 2A) lay within the ReaChR dynamic range as defined by the step response. Plots of mean firing rate across contrast revealed that both genotypes showed high amplitude modulations in firing at both irradiances (Figure 2C; shown separately for ON and OFF units in ReaChR Grm6 rd/rd). Moreover, contrast response relationships confirmed that both genotypes were able to track a wide range of contrasts at both backgrounds (Figure 2D). The implication of sensitivity normalization is supported by similarities in the contrast level that produced half-maximal response amplitude ($C_{50}$) in the face of the 10× difference in mean irradiance ($C_{50}$ for 16.6 vs. 15.6 log photons/cm$^2$/s = 51% and 46% for ReaChR Grm6 rd/rd ON, 49% and 43% for ReaChR Grm6 rd/rd OFF, 73% and 76% for ReaChR Brn3c rd/rd ON, respectively).

### RGC targeting reduces variability in response polarity and kinetics

Having established that RGC targeting using the ReaChR Brn3c rd/rd mouse supported robust visual responses with equivalent photosensitivity and sensitivity normalization to that of ReaChR Grm6 rd/rd, we turned to the question of whether the identity of the target cells would impact the visual code. Response latency in the retina was reduced in the ReaChR Brn3c rd/rd compared with ReaChR Grm6 rd/rd (Figure 3A, median latency for ON response to step = 43 ms for Grm6, 30 ms for Brn3c, U = 13.71, $p < 0.001$ at sub-saturating intensity 15.95 log photons/cm$^2$/s) consistent with introduction of ReaChR later in the visual pathway. A survey of responses to the step stimulus at single-unit resolution (Figures 3B and 3C) indicated a bias toward units meeting an objective classification of "ON" excitation in ReaChR Brn3c rd/rd relative to ReaChR Grm6 rd/rd retinas. To quantify the magnitude of this realignment we calculated an ON-OFF bias index for each unit (from −1 = OFF to 1 = ON), which confirmed a significant shift toward ON responses in ReaChR Brn3c rd/rd relative to ReaChR Grm6 rd/rd (Figure 3D, median ON-OFF bias index = 0.81 and 0.4, respectively, U = −15.57, $p < 0.001$). Indeed, units with OFF or biphasic ON/OFF responses were almost completely lacking from ReaChR Brn3c rd/rd retinas. We further quantified step responses in terms of their persistence. A transience index (from 0 = highly transient to 1 = highly sustained) revealed diversity in both genotypes (Figure 3E), but a significant bias toward more sustained responses in ReaChR Brn3c rd/rd (median = 0.25 for Grm6, 0.36 for Brn3c, U = −11.19, $p < 0.001$).

Some differences in step responses between genotypes were also apparent in the dLGN. Just as in the retina, response latency at sub-saturating irradiance (16.97 log photons/cm$^2$/s) was reduced in ReaChR Brn3c rd/rd compared with ReaChR Grm6 rd/rd dLGN (Figure 3F, median = 40 ms for Grm6, 23 ms for Brn3C, U = 7.01, $p < 0.001$). There remained significantly more variability in polarity in ReaChR Grm6 rd/rd compared with ReaChR Brn3c rd/rd dLGN units (Figures 3G and 3H), with the latter showing strong ON bias (median ON-OFF bias index = 0.32 for Grm6, 0.69 for Brn3C, U = −7.87, $p < 0.001$, Figure 3I). However, the genotype difference in response persistence was not found in the

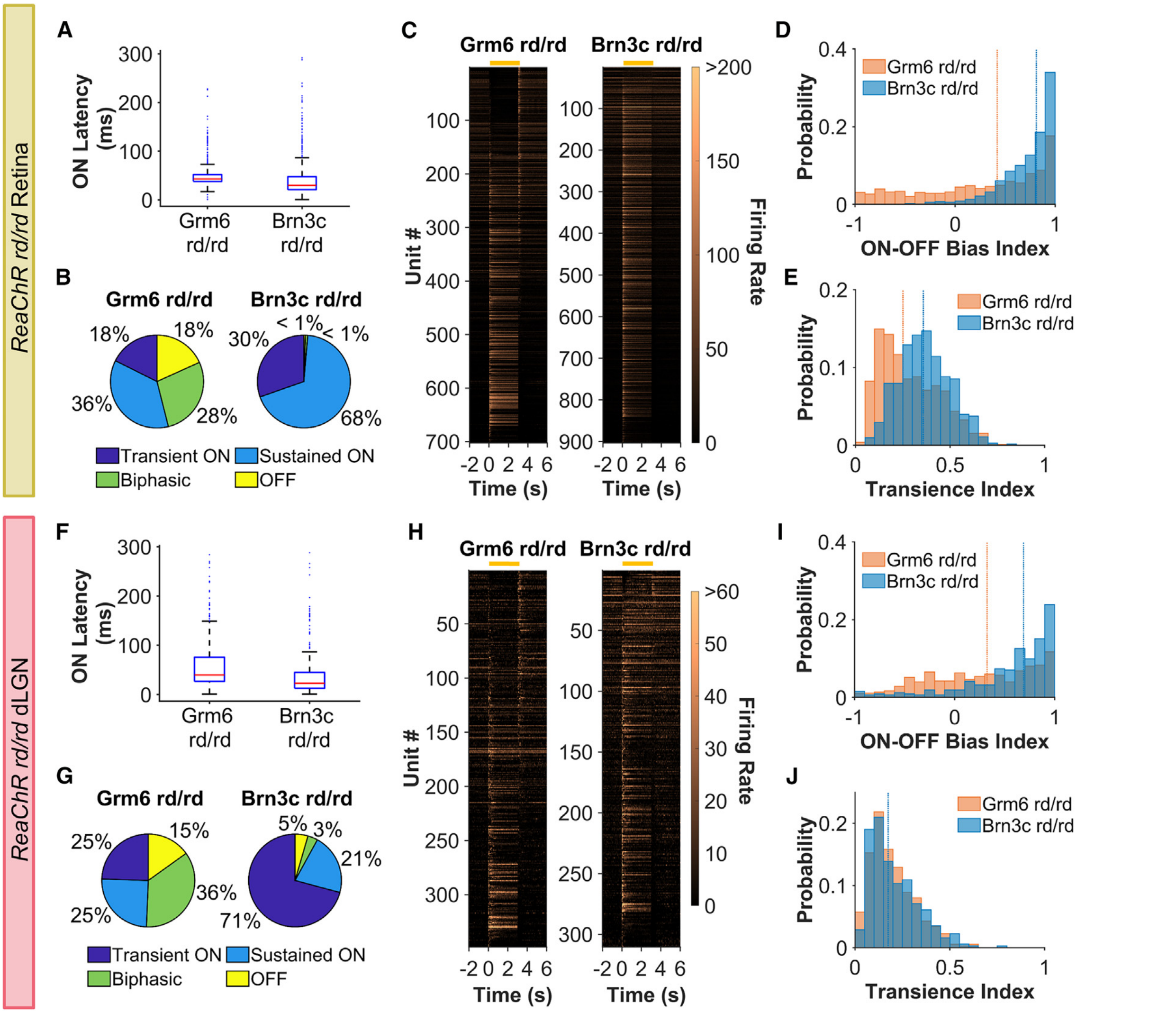


**Figure 3. Responses to step stimulus**

(A and F) Latency to onset of step for (A) retinal units ($n$= 560 for ReaChR Grm6 rd/rd and 787 for ReaChR Brn3c rd/rd) and (F) dLGN units ($n$ = 266 for ReaChR Grm6 rd/rd and 257 for ReaChR Brn3c rd/rd). (B and G) Response type classification. (C and H) Heatmap of mean PSTH for step stimulus (light on from 0 to 3 s) for LR units ordered by bias index from OFF (top) to ON (high). Each row represents an individual unit, yellow bar shows timing of step stimulus. (D and I) ON-OFF bias index. (E and J) Transience index. Data in (B)–(E) are from retinal units ($n$ = 702 for ReaChR Grm6 rd/rd and 903 for ReaChR Brn3c rd/rd) and in (G)–(J) from dLGN ($n$ = 348 for ReaChR Grm6 rd/rd and 310 for ReaChR Brn3c rd/rd).

dLGN, with both genotypes showing more transient responses in the brain (median transience index = 0.17 for both genotypes, U = −0.64, $p$ = 0.520, Figure 3J).

ReaChR Brn3c rd/rd retinas showed less diversity in response polarity and transience compared not only with ReaChR Grm6 rd/rd, but also with published reports of the intact retina.[40,41] This implies that ReaChR-driven activation is unable to recreate native visual response properties of many RGCs (e.g., those with highly transient and OFF or ON/OFF polarity). To address this possibility more directly, we carried out a series of MEA recordings in visually intact retinas from mice containing ReaChR expression under the *Brn3c* promoter that are also heterozygous for the *Pde6b* $^{rd1}$ mutation (termed ReaChR Brn3c rd/+ here, Figure 4A). In these animals, we were able to record both the native photoreceptor response (at light intensity below ReaChR threshold, 13.95 log photons/cm$^2$/s), and the isolated ReaChR responses (at high irradiance, 15.95 log photons/cm$^2$/s, following pharmacological blockade of rod and cone signaling). This allowed us to perform a within-unit comparison of the photoreceptor-driven (PRC-only) and ReaChR-driven (ReaChR-only) responses (see materials and methods for details, Figure 4B).

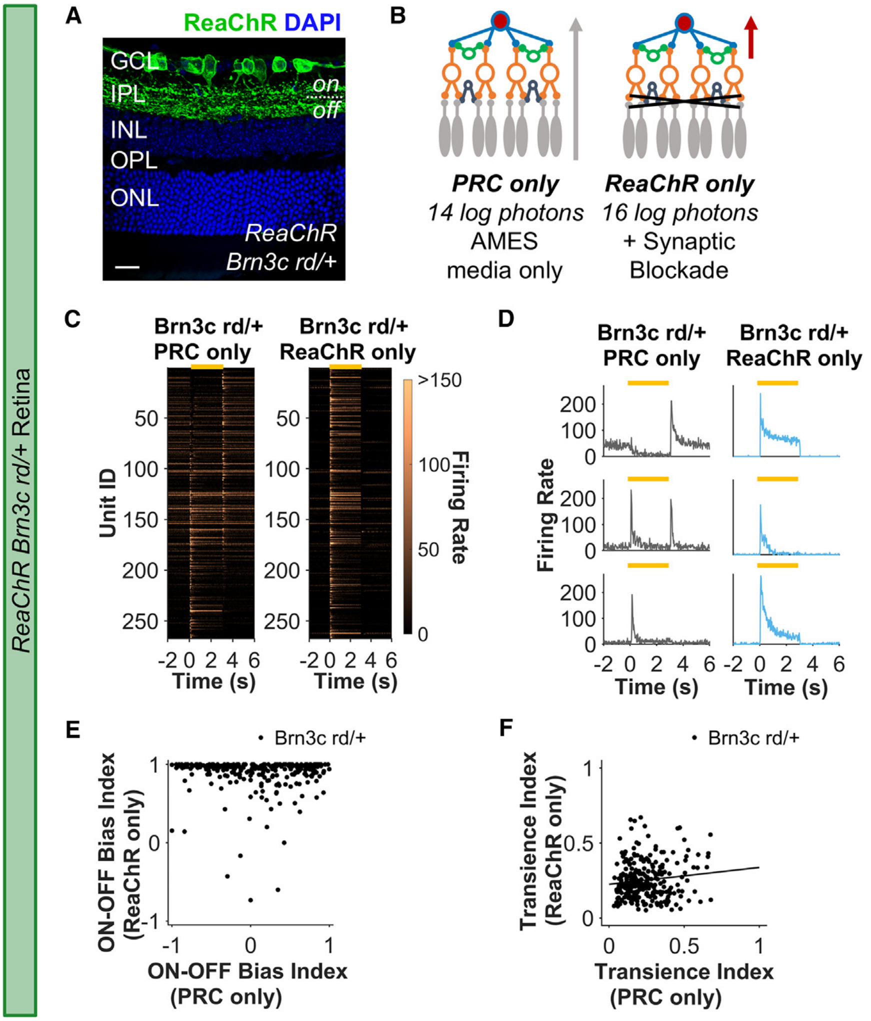


**Figure 4. Photoreceptor vs. ReaChR-driven activity in ReaChR Brn3c rd/+ retinas**

(A) Immunohistochemistry from retinal sections from ReaChR Brn3C rd/het stained for DAPI (blue) and ReaChR-mCitrine (green). Scale bar, 20 μm. (B) *Brn3c*-positive RGCs are identified from ReaChR Brn3c rd/+ recordings based on light response after synaptic blockade of rod/cone input. The photoreceptor-driven (PRC-only) responses recorded at lower light intensity, below threshold for ReaChR activation, in AMES medium were compared with ReaChR-driven responses recorded at bright intensities in AMES medium containing rod/cone blockers. (C) Heatmap of mean PSTH for step stimulus (light on from 0 to 3 s) for LR units ordered by bias index from OFF (top) to ON (high) in PRC-only condition. Each row in left and right heatmaps represents the same individual unit recorded under PRC-only (left) and ReaChR-only (right) conditions. (D) Example responses to step stimulus for three individual retinal units (in rows) recorded under PRC-only (left column) and ReaChR-only (right column) conditions. (E) ON-OFF bias index. (F) Transience index. Data in (C, E, and F) are from $n$ = 267 units from ReaChR Brn3c rd/+ (must be LR under both PRC and ReaChR-only conditions to be included). Yellow bar in (C) and (D) shows timing of step stimulus.

An overview of step responses suggested that, across the entire population, there was indeed more diversity in PRC-only compared with ReaChR-only responses (Figure 4C). Moreover, we identified many units that either switched polarity, lost OFF response components, or showed more sustained responses when activated via ReaChR compared with native photoreceptor input (Figures 4C and 4D). These could represent examples of units whose visual response properties are altered by ReaChR expression. However, native ganglion cell responses can also change as a function of background light intensity.[51]

Two lines of argument support the hypothesis that at least some of the differences in response properties between PRC and ReaChR conditions reflect a genuine disconnect between the native response of ganglion cells and that produced by direct optogenetic stimulation. Firstly, a unit-by-unit comparison confirms that a great deal of diversity in response polarity in the PRC-only condition is lost in the ReaChR-only condition (Figures 4D and 4E), with ReaChR-only responses showing very strong ON bias. Secondly, there was no significant relationship in ON-OFF bias index of individual units under the two conditions (Pearson R = −0.02, $p$ = 0.648, Figure 4E), which could be described by a flat line. Nevertheless, to more directly determine whether a reduction in response diversity is expected at high irradiance we recorded responses from WT retinas under the same two intensities (13.95 and 15.95 log photons/cm$^2$/s). These recordings showed equivalent diversity in ON-OFF bias at high vs. low irradiance and strong correlation in this parameter at single-unit level (Figures S1A and S1B, Pearson R = 0.69, $p$ < 0.001). Overall, these data confirm that at least some units that would be expected to have marked OFF response components instead become strongly ON biased under ReaChR stimulation.

ReaChR expression in RGCs likely contributed to the bias toward sustained responses seen in ReaChR Brn3c rd/rd, because there was weaker correlation in transience index between ReaChR vs. PRC conditions in ReaChR Brn3c rd/+ retinas (Figure 4F, Pearson R = 0.12, $p$ = 0.038) compared with high vs. low irradiance in WT retinas (Figure S1C, Pearson R = 0.42, $p$ < 0.001). Interestingly, however, a degree of inter-unit variation in transience was retained in the ReaChR-only condition and this was weakly correlated with transience in the PRC-only condition of ReaChR Brn3c rd/+ retinas (Pearson R = 0.12). These latter findings suggest that transience may be partly an intrinsic property of RGCs irrespective of whether they receive visual input from photoreceptors or ReaChR.

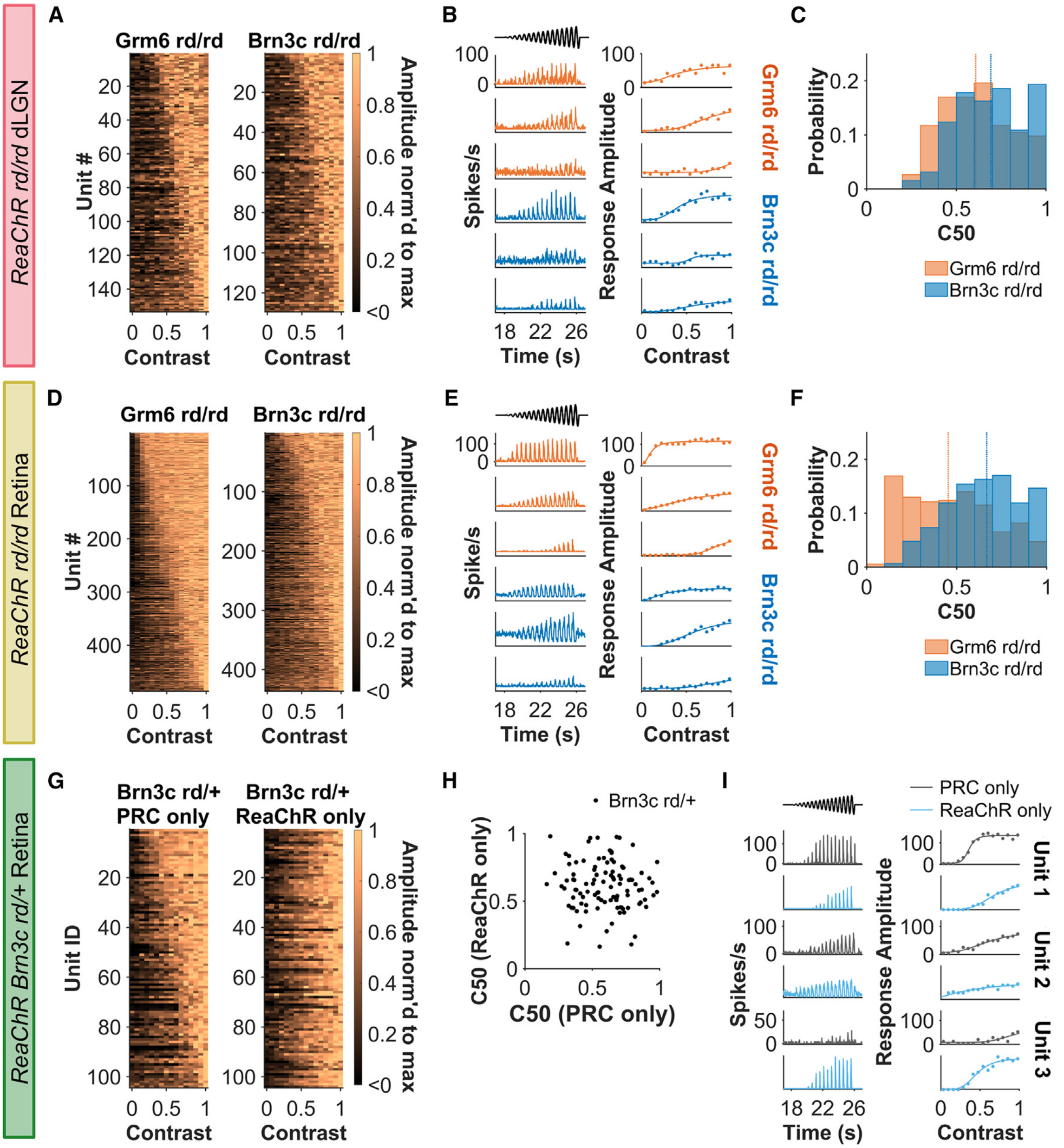


**Figure 5. Contrast sensitivity**

(A, D, and G) Heatmap of maximum normalized response amplitude across contrasts for LR units ordered by half-maximal contrast amplitude ($C_{50}$) from most (top) to least sensitive (bottom). Each row represents an individual unit and in (G) each row in left and right heatmaps represents the same unit recorded under different conditions (ranked based on $C_{50}$ for PRC-only). (B, E, and I) Example firing rate to contrast chirp (left column) and contrast sensitivity function (right column) for three representative units. Data shown in (I) are grouped by rows to show same unit under PRC- and ReaChR-only conditions. Timing of contrast chirp stimulus shown in black. (C, F, and H) $C_{50}$ derived from best-fit contrast response function. Scatterplot in (H) shows $C_{50}$ values derived from same unit under different conditions. Data in (A)–(C) are from dLGN units (*n* = 153 for ReaChR Grm6 rd/rd and 129 for ReaChR Brn3C rd/rd), (D)–(F) from rd/rd retina (*n* = 485 for ReaChR Grm6 rd/rd and 436 for ReaChR Brn3C rd/rd), and (G)–(I) from ReaChR Brn3c rd/+ retina (*n* = 104 units). Contrast function must have $R^2$ > 0.5 and LR units must have spiking in >10% bins during contrast chirp to be included.

### High-contrast sensitivity units lacking in Brn3c RGC targeting

We next examined how well each of the target cell types for optogenetic therapy recreated diversity in contrast sensitivity. In the dLGN, we saw a range of responses to the contrast chirp in both ReaChR Brn3c rd/rd and ReaChR Grm6 rd/rd animals (Figure 5A). This included examples of units showing graded increases in response across the contrast range; saturating responses at intermediate contrast; or responses to only the highest contrasts presented

(Figure 5B). Extracting $C_{50}$ from Naka-Rushton functions fit to contrast responses revealed that the ReaChR Grm6 rd/rd retinal units were biased to slightly higher contrast sensitivity compared with ReaChR Brn3c rd/rd (median $C_{50}$ = 61% for Grm6 and 69% for Brn3c, U = −3.20, $p$ = 0.001, Figure 5C). The variation in contrast response was even more apparent in the retina (Figure 5D), with some units demonstrating large amplitude responses even at relatively low contrasts (Figure 5E). Comparison of $C_{50}$ values revealed that such high contrast sensitivity was a particular property of the ReaChR Grm6 rd/rd retina (Figure 5F) and, accordingly, ReaChR Brn3c rd/rd units on average had significantly reduced contrast sensitivity (median $C_{50}$ = 45% for Grm6 and 67% for Brn3c, U = −11.13, $p$ < 0.001).

ReaChR Brn3c rd/rd retinas appear to lack high-contrast sensitive units—but does this indicate a fundamental inability of direct optogenetic RGC activation to produce high contrast sensitivity? To address this question, we turned to comparing PRC- and ReaChR-only responses in visually intact ReaChR Brn3c rd/+ animals (see above, Figure 5G). This revealed that high contrast sensitivity ($C_{50}$ < 0.5) units were rare under both PRC and ReaChR conditions (Figure 5H). The most straightforward explanation then is that high contrast sensitivity is rare in *Brn3c*-positive ganglion cells. Further to the conclusion that ReaChR itself does not introduce a bias in contrast sensitivity, there was a spread of $C_{50}$ values >0.5 in both conditions (Figure 5H) and it was possible to identify units showing both increased and decreased contrast sensitivity in ReaChR vs. PRC conditions (Figure 5I).

Turning to the question of whether ReaChR was able to recapitulate native contrast sensitivity of individual units, there was no significant correlation between $C_{50}$ under ReaChR and PRC conditions in ReaChR Brn3c rd/+ retinas (Pearson R = −0.02, $p$ = 0.800, Figure 5H). However, analysis of this parameter in WT retinas under the two irradiances used for ReaChR-only and PRC-only conditions revealed only a weak correlation (Pearson R = 0.15, $p$ = 0.01, Figure S2). It seems then that, under these conditions, the $C_{50}$ of individual units shows substantial plasticity over changes in irradiance, making it impossible for us to determine the extent to which the ReaChR-driven response recapitulates native contrast response properties at single-unit level.

#### Temporal frequency tuning is more stereotyped with RGC targeting

We found that dLGN units responded across the frequency range (1–8 Hz) of the temporal chirp in both ReaChR Brn3C rd/rd and ReaChR Grm6 rd/rd (Figure 6A). This encompassed units with broad and narrow temporal frequency tuning (Figure 6B). The preferred temporal frequency (peak TF) for each unit was extracted by fitting a half-Gaussian function to the average response amplitude at each temporal frequency. Peak TF was slightly higher across ReaChR Brn3C rd/rd than ReaChR Grm6 rd/rd units (median peak TF = 1.72 Hz for Grm6, 2.16 Hz for Brn3c, U = −7.16, $p$ < 0.001, Figure 6C). A more notable effect was a reduction in diversity in this parameter in ReaChR Brn3C rd/rd (Figure 6D, D = 0.33, $p$ < 0.001, Kolmogorov-Smirnov test). This revealed that, in the dLGN, ReaChR Grm6 rd/rd units had more variety in their temporal frequency tuning profiles (Figure 6A), including low-pass and bandpass units (Figure 6B), while the ReaChR Brn3C rd/rd dLGN was dominated by units with bandpass tuning with peak TF at 2 Hz.

Temporal frequency tuning was more diverse in the retina than the dLGN (Figure 6E). We found individual ReaChR Grm6 rd/rd retinal units (Figure 6F) with low-pass tuning, bandpass tuning, or strong responses at all temporal frequencies. In comparison, ReaChR Brn3c rd/rd units were biased to 2 Hz bandpass tuning, as in the dLGN. Thus, although there was no significant difference in calculated peak TF between genotypes (median peak TF = 1.79 for Grm6, 1.93 for Brn3c, U = −0.26, $p$ = 0.796, Figure 6G), there was a difference in cumulative distribution—with ReaChR Brn3c rd/rd units more biased to 2 Hz (Figure 6H, D = 0.16, $p$ < 0.001, Kolmogorov-Smirnov test).

Examination of ReaChR Brn3c rd/+ responses suggests that the 2 Hz bias is a property of ReaChR in RGCs. Thus, RGCs with diverse tuning profiles during photoreceptor-driven conditions realign toward 2 Hz bandpass tuning under ReaChR-driven conditions (Figures 6I–6L) to leave no statistically significant relationship between peak TF under ReaChR vs. PRC conditions (Figure 6L, Pearson R = −0.01, $p$ = 0.918). Conversely, no such realignment was apparent in WT retinas under the same two light intensities (Figure S3, Pearson R = 0.26, $p$ < 0.001).

#### RGC targeting produces an impoverished visual code

Having examined how targeting BC vs. RGCs affects individual visual response properties, we turned to a more holistic examination of the visual code. Applying community detection, we aimed to group units based on sparse principal components (sPCs) analysis of their response to the entire chirp stimulus. This approach identifies information channels defined by their response across different stimulus features. By pooling the ReaChR Brn3c and Grm6 rd/rd data with that from WT (photoreceptor-driven) retinas and then performing clustering and community detection, we hope to determine whether the optogenetic interventions: (1) differed in the number of parallel information channels recreated, (2) introduced bias in how units were distributed across these channels, and (3) closely recreated feature selectivity combinations found in WT mice.

Starting with the retina, nine communities were identified across the three genotypes (Figures 7A–7C). ReaChR Grm6 rd/rd and WT units were distributed across all nine communities, consistent with our previous report that ReaChR expression in ON BCs can recapitulate much of the diversity of the WT visual code.[42] By contrast, ReaChR Brn3c rd/rd units appeared in fewer communities, being primarily restricted to communities 6, 7, and 8 (characterized by sustained ON responses, bandpass temporal tuning, and intermediate contrast sensitivity). RGC subtypes with these response properties include ON sustained and ON alpha RGCs (such as groups 22–24 in Baden et al.[40]). Only 1 Brn3c unit was found in

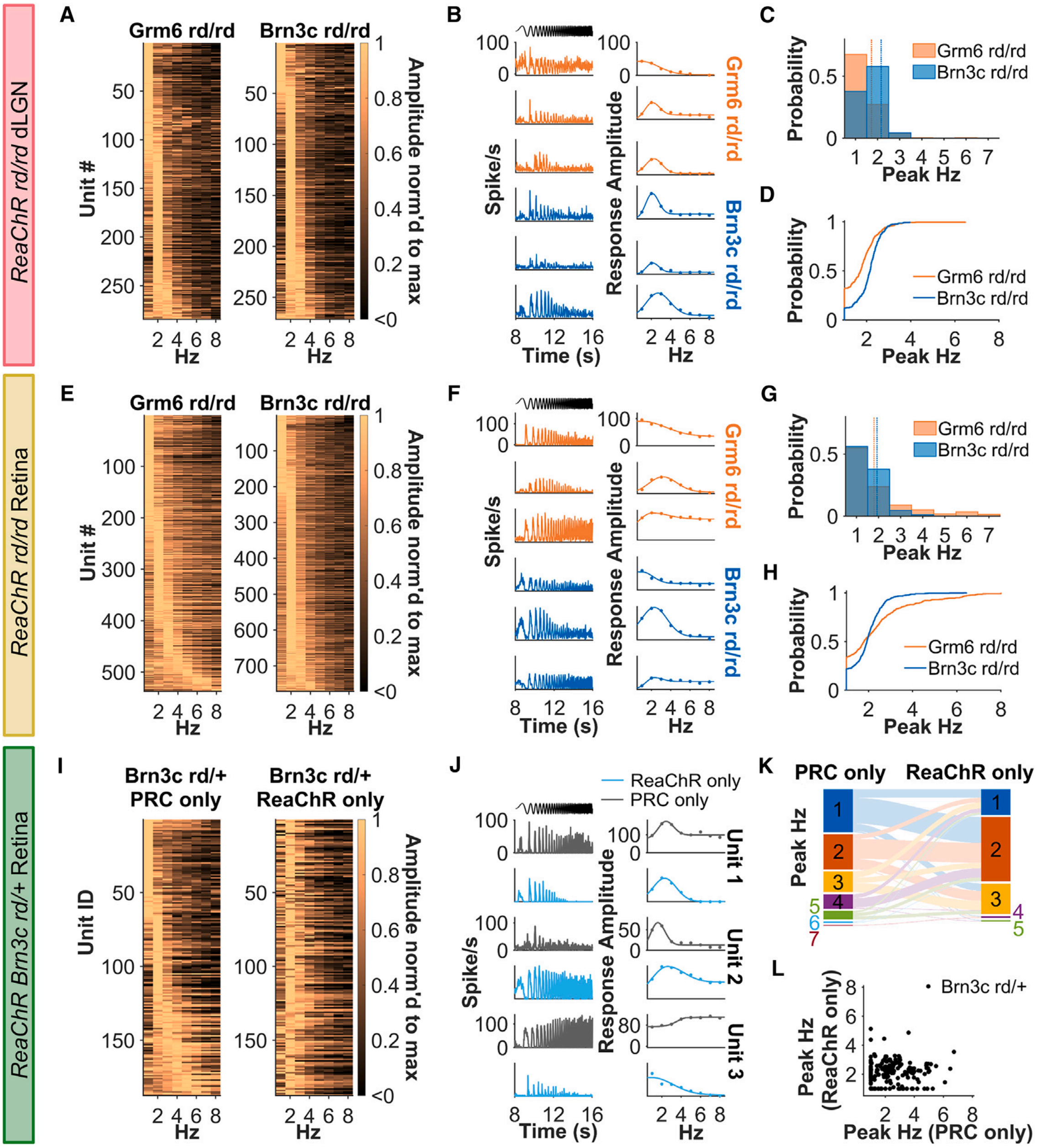


**Figure 6. Temporal frequency tuning**

(A, E, and I) Heatmap of maximum normalized response amplitude across average frequency (Hz) for LR units ordered by peak temporal frequency (peak TF) from low (top) to high frequency (bottom). Each row represents an individual unit and in (I) each row in left and right heatmaps represents the same unit recorded under different conditions (ranked based on peak TF for PRC-only). (B, F, and J) Example firing rate to contrast chirp (left column) and contrast sensitivity function (right column) for three representative units. Data shown in (J) are grouped by rows to show same unit under PRC- and ReaChR-only conditions. Timing of temporal chirp stimulus shown in black. (C and G) Histogram and (D and H) cumulative distribution function for peak temporal frequency (peak Hz) derived from best-fit temporal tuning function. (K) Sankey diagram showing peak temporal frequency (peak Hz) of units for PRC- and ReaChR-only conditions. Scatterplot in (L) shows peak TF values derived from same ReaChR Brn3c rd/+ retinal unit under different conditions. Data in (A)–(D) are from dLGN units (*n* = 285 for ReaChR Grm6 rd/rd and 270 for ReaChR Brn3C rd/rd), (E)–(H) from rd/rd retina units (*n* = 285 for ReaChR Grm6 rd/rd and 270 for ReaChR Brn3c rd/rd) and (I)–(L) from Brn3c rd/+ retina units (*n* = 187). Best-fit temporal tuning profiles must have $R^2 > 0.5$, LR units must have spiking in >10% bins for contrast chirp, Gaussian spread >0.51 (defined by at least three points) to be included.

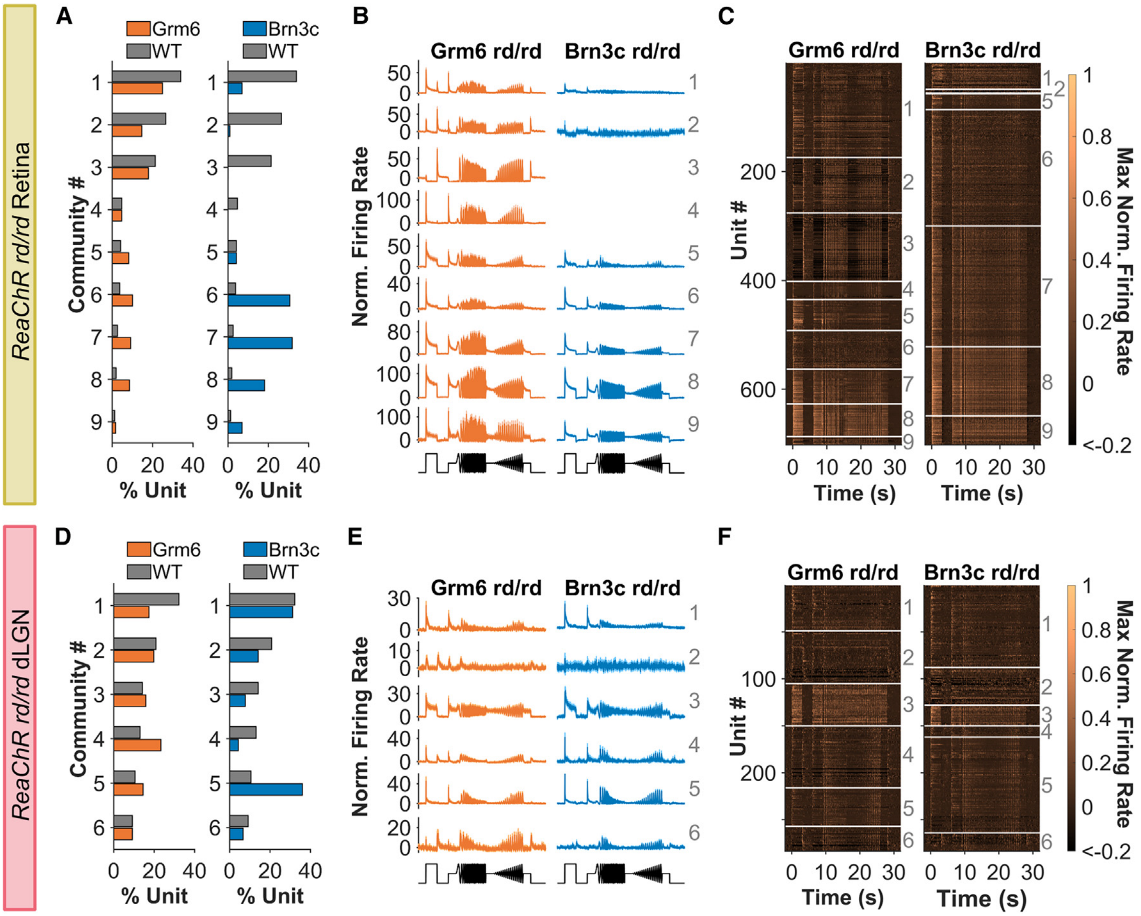


**Figure 7. Visual code in ReaChR rd/rd mice**

(A and D) Distribution of (A) retina ($n$ = 702 units per genotype) and (D) dLGN units ($n$ = 283 units per genotype) across communities—downsampled to get same number of units in each genotype. (B and E) Mean firing rate for units from each community (numbered in gray on right) in (B) retina and (E) dLGN. Data from each genotype is shown for communities with sufficient units to calculate mean ($n \geq 3$). (C and F) Heatmap showing mean PSTH for individual units (each row) in each community. Boundaries of each community are shown with white lines and community identity is shown by gray number on right for (C) retina and (F) dLGN from rd/rd mice. Data from ReaChR Grm6 rd/rd mice in orange, ReaChR Brn3c rd/rd in blue, and WT mice in gray. Labels for different communities are shown in gray text in (B), (C), (E), and (F).

community 3 (OFF units with high contrast sensitivity and minimal temporal tuning), which has similar response profile to OFF sustained and OFF alpha RGCs (e.g., groups 7–9 in Baden et al.[40]). No Brn3c units were in community 4 (highly transient ON responses with high-pass temporal tuning and intermediate contrast sensitivity), response properties similar to ON transient RGCs (e.g., groups 18–20 in Baden et al.[40]). The implication is that the method of optogenetic targeting had impacted the visual code, and indeed a shuffle test revealed that the distribution of units across communities was significantly different between ReaChR Grm6 and Brn3c rd/rd retinas ($p$ = 0.001). While there was a small but significant difference in the distribution of units from WT and ReaChR Grm6 rd/rd retinas across communities ($p$ = 0.025) as reported previously,[42] the differences from WT were more stark in the ReaChR Brn3c rd/rd retina ($p$ < 0.001), with Brn3c units found mostly in communities (6–8) sparsely represented in WT retina. Community detection analysis of PRC-driven and ReaChR-driven responses in Brn3c rd/+ retina showed this was due to a shift in the visual features encoded by individual units (Figure S4).

A similar pattern was observed in the dLGN, where we found 6 communities (Figures 7D–7F). As in the retina, the distribution of units across these communities was significantly different between genotypes ($p$ < 0.005)—with ReaChR Brn3c rd/rd units concentrated in communities 1 and 5 (transient ON with bandpass temporal tuning and intermediate contrast sensitivity), while ReaChR Grm6 rd/rd units were more evenly distributed across the different response categories, suggesting that the more diverse visual code produced by ON BC targeting persists at higher levels of visual projection. As in the retina, the ReaChR Grm6 rd/rd responses more reliably recreated the visual code seen in WT animals, with similar distribution of units across communities ($p$ = 0.154, shuffle test), while this difference

**Table 1. Population-level changes in visual response properties for ON BC and RGC optogenetic targeting**

| | ReaChR in RGCs compared with ON BC (population-level) | |
|---|---|---|
| Visual response property | Retina | dLGN |
| Photosensitivity | similar in both | – |
| Maximum firing rate | lower in RGC | similar in both |
| Response reproducibility | lower in RGC | lower in RGC |
| Sensitivity normalization | present and similar in both | – |
| Response onset latency | shorter in RGC | shorter in RGC |
| Response polarity | more ON-biased in RGC (missing OFF/biphasic units) | more ON-biased in RGC |
| Response transience | more sustained in RGC (missing highly transient units) | similar in both |
| Contrast sensitivity | less sensitive in RGC (missing units with high contrast sensitivity) | less sensitive in RGC |
| Temporal frequency tuning | more stereotyped in RGC (missing units with low- or high-pass tuning) | more stereotyped and biased to higher frequencies in RGC |
| Diversity of visual responses | reduced in RGC | similar in both |
| Visual code compared with WT | less WT-like in RGC | less WT-like in RGC |

between WT and Brn3c responses was more exaggerated ($p$ = 0.009, shuffle test).

## DISCUSSION

Our aim was to provide a close comparison of therapeutic efficacy for retinal ganglion vs. ON BC expression in optogenetic vision restoration. To this end we compared visual responses in transgenic mouse lines expressing the same optogenetic actuator (ReaChR) under the same promoter in targeted cell types across the retina, minimizing the variation in the spatial distribution, density, and extent of expression that is a feature of viral gene delivery methods. Our findings (summarized in Table 1) confirm that ON BCs provide clear advantages in terms of the quality of restored visual code. ON BC targeting did not impair sensitivity as reported previously[27,29] and, although response latency was increased compared with RGC targeting, it remained within the range for intact vision. ON BC targeting produced higher trial-to-trial reproducibility, but its most significant advantage lay in the richness of the restored visual code. Whereas ReaChR Brn3c rd/rd units converged to a relatively stereotyped response profile in terms of polarity, transience, temporal frequency tuning, and contrast sensitivity, all of these characteristics showed greater variability in ReaChR Grm6 rd/rd. As a result, the ReaChR transgenic line with ON BC targeting better approached the diversity of visual information channels in the intact retina.

Some aspects of the ON BC advantage are consistent with *a priori* expectations based upon known retinal physiology. Visual signals introduced to ON BCs must traverse the retina in order to reach ganglion cells and the brain. In doing so, they may benefit from the information-processing capacity of the inner retina in a way that visual signals introduced at the ganglion cell level cannot. The most obvious impact of such processing is the appearance of OFF and ON/OFF responses in ReaChR Grm6 rd/rd mice. The primary ReaChR response (cation conductance) should be the same in both ON bipolar and RGCs, but while the resultant light-dependent depolarization can only appear as an ON excitation when introduced to RGCs, it can be transformed to OFF responses downstream of ON BCs thanks to crossover inhibition with the retinal OFF pathway via AII amacrine circuitry.[52–58] Similarly, temporal frequency tuning is known to be influenced both by intrinsic properties of ON BCs[59] and circuit mechanisms in the inner plexiform layer,[60–62] providing plausible explanations for enhanced diversity in this characteristic in ReaChR Grm6 rd/rd mice. Diversity in response transience is also a property of neurons upstream of RGCs[63] and our data are consistent with the view that introducing ReaChR in ON BCs allows more of that variability in response persistence to be recovered. Interestingly, however, the retention of a weak but significant correlation between this property in PRC and ReaChR-driven responses in the intact ReaChR Brn3c retina indicates that transience is defined to some extent in RGCs in such a way as to be accessible to direct optogenetic activation.

The more stereotyped responses of ReaChR Brn3c rd/rd imply a problem for the visual code in this genotype beyond its reduced diversity. Comparison of ReaChR and native photoreceptor-driven responses in visually intact rd/+ mice reveal that, in many cases, the fundamental sensory response properties of units are different under direct optogenetic activation. Many OFF units switch to ON polarity and there are quantitative changes in response transience, contrast sensitivity and temporal frequency tuning. In this way, firing patterns at an individual unit level convey quite different information about the visual scene under direct ReaChR activation. The extent to which this presents a problem for downstream processing remains uncertain and it is worth highlighting that some visual properties, such as response polarity, show substantial natural plasticity.[51,64,65] That plasticity is particularly reported across changes in irradiance, and we have included control recordings comparing response property changes within units between irradiances in WT mice. Shifts in most response parameters, including response polarity, transience,

and temporal tuning, were more common when switching from photoreceptor to optogenetic activation than when adjusting irradiance, confirming that the normal visual code is disrupted by ReaChR expression in RGCs beyond what is expected for natural plasticity. The exception is contrast sensitivity, which also shows large changes at a single-unit level across irradiances, making it impossible to determine whether ReaChR in RGCs imposes additional alterations to diversity in this parameter.

Another theoretical advantage of ON BC targeting is that it may provide access to all retinal output channels in a way that may be hard to achieve for RGC targeting, given cell-type specificity of viral transduction efficiency.[26] The Brn3c Cre line provides an example of this challenge as it targets the subset of RGCs that are *Brn3c* positive,[48] comprising 27 of the 42 RGC functional subtypes identified in Goetz et al.[41] Our parallel recordings of ReaChR and photoreceptor-derived responses in rd/+ mice provide an insight into the degree to which the incomplete coverage of RGC types contributes to the reduced visual code diversity in ReaChR Brn3c rd/rd. We find that the Brn3c-positive cells show diversity in response polarity, transience, and temporal frequency tuning under photoreceptor driven conditions, confirming that the reduced diversity in these properties cannot be attributed solely to limitations in ganglion cell coverage. However, high contrast sensitivity cells do appear missing from the Brn3c population suggesting that incomplete coverage of the RGC types contributes to the reduced diversity in this property in ReaChR Brn3c rd/rd mice.

Although our data reveal several advantages of ON BC targeting, it is important to recognize that they do also provide encouragement for ganglion cell targeting. There may be good practical reasons for targeting ganglion cells in clinical practice including difficulties in efficiently transducing ON BCs and the challenges of progressive inner retinal degeneration (although see Rodgers and co-workers[42,66]). Moreover, the benefits of ON BC targeting may be less realized in slower forms of degeneration or conditions in which BC function itself is impaired.[37,67–69] Our data extend the existing evidence that targeting ganglion cells can provide visual signals with helpful characteristics, such as a high spatiotemporal resolution.[22] We found widespread and reproducible visual responses in ReaChR Brn3c mice across a range of irradiances, contrasts, and temporal frequencies. ReaChR Brn3c rd/rd also retain some diversity in response properties. Although strongly biased toward ON responses, there are a few rare examples of OFF excitation in the dLGN recordings. More encouragingly, single units in both retina and dLGN show surprising diversity in other visual response parameters. Previous reports have seen a wide range of response transience following RGC-biased viral delivery[20,22,43] and we confirm that this is also the case when ReaChR expression is restricted to RGCs.[25] We further show variability in contrast sensitivity, as well as two types of temporal frequency tuning profiles in the retina during RGC optogenetic targeting (low pass and 2 Hz bandpass). These findings highlight an interesting question of the extent to which, in normal vision, such properties are defined by intrinsic properties of individual RGCs vs. the upstream circuit. Indeed, intrinsic mechanisms shaping visual feature extraction, such as contrast sensitivity, are beginning to be described.[70] From a practical perspective, these properties contribute to a richer visual code following RGC targeting than may have been initially anticipated. This could be further enhanced with focal light stimulation combined with pre-processing of the image to match the light stimulation to response properties of the targeted RGC. The addition of focal stimulation might mean that targeting ganglion cells could provide a better outcome.[71]

An important property shared by ON bipolar and ganglion cell interventions was sensitivity normalization. Natural photoreceptors face the challenge of encoding small local modulations in light intensity across big differences in background light with a variety of adaptation mechanisms. A reasonable concern with optogenetic interventions is that, absent such mechanisms, restored vision would have a narrow sensitivity range, saturating at bright backgrounds. In fact, we find evidence of sensitivity normalization in both ReaChR Grm6 rd/rd and ReaChR Brn3c rd/rd, with contrast sensitivity retained across brighter irradiances. Inner-retinal mechanisms of light adaptation may contribute to this ability in ReaChR Grm6 rd/rd,[72–76] but the ReaChR Brn3c rd/rd data imply that it may also be an intrinsic property of ReaChR activity and/or host cell physiology.

The decision of which cell types to target for optogenetic therapy in clinical application will be a complex one and may encompass patient-specific considerations such as inner retinal integrity as well as practical challenges of viral gene delivery.[77] Our side-by-side comparison of the quality of restored vision from ON bipolar vs. RGC targeting suggests that the theoretical advantages of introducing visual signals as early as possible to the circuit are indeed apparent in a richer visual code. However, they also confirm an impressive ability to encode dynamic visual stimuli across a range of irradiances with either targeting method and thus provide general encouragement for this therapeutic avenue.

## MATERIALS AND METHODS

This study includes electrophysiology data from units in ReaChR Grm6 rd/rd and visually intact mice previously analyzed in Rodgers et al.[42] For retinal recordings, this original dataset has not been altered, while for the dLGN dataset additional recordings from ReaChR Grm6 rd/rd mice have been added. All data from ReaChR Brn3c rd/+ or rd/rd mice was newly generated for this study and has not been published previously. Existing data from ReaChR Grm6 rd/rd and WT mice were analyzed, alongside new recordings from ReaChR Brn3c animals, using new criteria to identify light responsive units.

### Animals

MEA recordings were from: 5 retinas from 5 *ReaChR Grm6*$^{Cre/WT}$ *Pde6b*$^{rd1/rd1}$ mice (4 females, 1 male, 144–169 days old); 7 retinas from 4 *ReaChR Brn3c*$^{Cre/WT}$ *Pde6b*$^{rd1/rd1}$ mice (2 males, 2 females, 153–180 days old); 5 retinas from 4 *ReaChR Brn3c*$^{Cre/WT}$ *Pde6b*$^{rd1/WT}$ mice (1 male, 3 females, 186–228 days old). MEA recordings from

visually intact animals were from: 3 retinas from 3 ReaChR Grm6$^{WT/WT}$ Pde6b$^{rd1/WT}$ mice (2 females, 1 male at 144–158 days old); 9 retinas from 8 *C57Bl/6* mice (Envigo, 6 females, 2 males at 150–164 days old) as described in Rodgers et al.[42] LGN recordings were from: 16 placements from 7 *ReaChR Grm6$^{Cre/WT}$ Pde6b$^{rd1/rd1}$* mice (3 females, 4 males, 150–166 days old); 14 placements from 6 mice from *ReaChR Brn3c$^{Cre/WT}$ Pde6b$^{rd1/rd1}$* mice (4 females, 2 males, 153–175 days old). LGN recordings from visually intact animals were from 11 placements from 4 *C57Bl/6* mice (University of Manchester, 3 females, 1 male, 141–196 days old) as described in Rodgers et al.[42] *ReaChR Grm6$^{Cre}$ Pde6b$^{rd1}$* mice were produced at University of Oxford, *ReaChR Brn3c$^{Cre}$ Pde6b$^{rd1}$* mice were produced at University of Manchester. All animals were given water and food *ad libitum*, kept under 12:12 light-dark cycle and group housed. Home cage lighting intensity was below threshold for ReaChR activation. All experiments were conducted in accordance with the Animals Scientific Procedures Act of 1986 (United Kingdom) and approved by ethical review committees at University of Oxford and University of Manchester.

### Transgenic mice

This study uses the ReaChR Grm6 rd strain, previously described in Rodgers et al.,[42] created by breeding Grm6$^{Cre/WT}$ (MGI: 4411993,[47] kindly shared by Robert Duvoisin, Oregon Health and Science University, USA) with ReaChR-mCitrine mice (MGI: 5605725) obtained from Jackson Laboratory (no. 026294). ReaChR Grm6 rd/rd mice were bred to be homozygous for ReaChR-mCitrine, heterozygous for Grm6 Cre and homozygous for Pde6b rd1. We also produced a new transgenic line—ReaChR Brn3c rd. Brn3c$^{Cre/WT}$ mice (MGI: 7470766,[48] kindly shared by Tudor Badea, National Eye Institute, NIH, USA) were bred with homozygous *ReaChR Grm6$^{WT/WT}$ Pde6b$^{rd1/rd1}$* from the *ReaChR Grm6 rd* colony maintained at the University of Manchester. Mice were bred to be homozygous for ReaChR, heterozygous for Brn3c Cre and either heterozygous (ReaChR Brn3c rd/+) or homozygous (ReaChR Brn3c rd/rd) for *Pde6b$^{rd1}$*. These mice express floxed *ReaChR-mCitrine* transgene in *Brn3c*-positive RGCs which express Cre recombinase. *Brn3c*-positive RGC subtypes have been defined based on morphology and functional properties[48] and include (Eyewire category[78] given in parentheses): ON-OFF direction selective (37c, 37d, 37r, 37v), ON orientation selective (72, 81i, 81o, 82wi, 82wo), suppressed-by-contrast (27, 2o), motion-sensitive (5to), local edge detectors (51), OFF horizontal orientation selective (2aw), and OFF sustained alpha (1wt). Genotyping was performed using primers to amplify Brn3c Cre (Fwd = 5′-CCGGGGTATAA ATGCTGTGG, Rev = 5′-CCTCATCACTCGTTGCATCG, 411 bp band), ReaChR and rd1 alleles (as described in Rodgers et al.[42]) or using an external genotyping service (Transnetyx). At 5 months old, *Pde6b$^{rd1/WT}$* mice are retinally degenerate, with complete loss of rods and the majority of cone photoreceptors. *Pde6b$^{rd1/WT}$* are visually intact and possess rod and cone photoreceptors. Degeneration occurs rapidly in the *rd1* mice, with the majority of photoreceptors lost before mice are 1 month old. In Cre-positive mice, the ReaChR-mCitrine transgene can be expressed from earliest expression of the relevant driver in eye development (Grm6 or Brn3c) and throughout the lifetime of the animal. Unless stated otherwise, mice are on a mixed C57Bl/6 × C3H background.

### Immunohistochemistry

Retinal sections were immunostained as described previously.[27,79] The mCitrine tag of ReaChR-mCitrine transgene was labeled using chicken anti-GFP polyclonal primary antibody (1:1,000, GFP-1020, AVES labs). Donkey anti-chicken 488 (1:250, T03-545-155, Jackson Immunoresearch) was used as secondary antibody. Fluorescence images were acquired using inverted LSM 710 laser scanning confocal microscope (Zeiss) with Zen 2009 image acquisition software (Zeiss). Individual channels were collected sequentially. Excitation laser lines were 405 and 488 nm with emission at 440–480 and 505–550, respectively. z Stack was acquired using an ×40 objective, with images collected every 1 μm in z axis. Global enhancement of brightness and contrast were applied to maximum intensity projection using ZenLite 2011 software (Zeiss).

### *In vivo* electrophysiology

Mice were anesthetized using urethane (intraperitoneal injection, 1.4–1.5 g/kg) and placed in a stereotaxic frame. An incision was made through scalp to expose the surface of the skull. A small hole was drilled 2.2 mm lateral and 2.2 mm posterior from bregma. The contralateral pupil was dilated using 1% atropine in saline (Sigma-Aldrich) and kept lubricated using mineral oil or Lubrithal (Dechra). Multi-electrode arrays (A4x16-Poly2-5mm-23s-200-177-A64, NeuroNexus) were coated in CM-DiI (Fisher Scientific); positioned at 2.2 mm lateral and 2.2 mm posterior relative to bregma and inserted to a depth of 2.5–3 mm to target LGN, confirmed by the presence of light responsive units to short steps of white light (2–5 s ON, 10 s OFF, 10 repeats; 16 log photons/cm$^2$/s). Once light responses were identified, mice were dark adapted for 20–30 min, allowing neuron activity to stabilize. Signals were acquired using the Recorder 64 system (Plexon), amplified (×3,000), high-pass filtered at 300 Hz, digitized at 40 kHz, and stored in 16 bit continuous format. Some additional recordings were performed by raising recording probe and moving 0.2 mm posterior or anterior before re-inserting into the dLGN. After experiments were complete, mice were killed by cervical dislocation and brains were fixed in 4% paraformaldehyde. Single-unit activity was isolated using Kilosort[80] and manually checked in Offline sorter (Plexon).

### Retinal MEA recordings

Mice were culled by cervical dislocation, enucleated, and retinas dissected under dim red light. Retinas were positioned ganglion-cell side down in MEA chambers (Multi Channel Systems) containing 252 electrodes (30 μm in diameter, spaced 100 μm apart). MEAs were then inserted in the MEA2100-256 recording system (Multi Channel Systems) and positioned in the light path of an inverted Olympus IX71 microscope. Retinas were kept at 34°C and were continuously perfused with AMES medium gassed with 95% $O_2$ and 5% $CO_2$. Neural signals were collected, amplified, and digitized at 25 kHz using MCS Experimenter software (Multi Channel Systems). Retina were dark adapted for at least 30 min before stimuli

presentation to allow neural activity to stabilize. Single units were isolated from retinal MEA data using SpikeSorter software (version 4.77b Nicholas Swindale, UBC). Raw data were filtered using a high-pass 4-pole 500 Hz Butterworth filter. Event detection was based on 4–5× median noise signal, with window width of 0.24 ms. Automatic spike sorting results were manually checked using SpikeSorter software and Offline Sorter (Plexon).

For ReaChR Brn3c rd/+ retinal recordings, retinas were first perfused with AMES medium during initial stimulus presentation before being perfused AMES with synaptic blockers during second round of stimuli. The following pharmacological blockade was used to isolate ReaChR-driven responses in Brn3c-positive ganglion cells: 100 μm L(+)-2-amino-4-phosphonobutyrate (group III metabotropic glutamate receptor agonist), 40 μm 6,7-dinitroquinoxaline-2,3-dione (AMPA/kainate receptor antagonist), and 30 μm d-2-amino-5-phosphonovalerate (NMDA receptor antagonist, Tocris).

#### Visual stimuli

For *in vivo* electrophysiology experiments, light was delivered using a CoolLED pE-4000 light source via a liquid light guide connected to a diffuser (Edmund Optics). This was positioned ∼5 mm from the eye contralateral to the hemisphere containing the recording site. White light was used for all stimuli (output from four LEDs at 385, 470, 550, and 660 nm). Neutral density filters were inserted in the light path to produce an intensity range from 12.99 to 16.97 effective log photons/$cm^2$/s.

For retinal MEA experiments, light was delivered using a white LED light source with daylight spectrum (ThorLabs, SOLIS-3C), with stimuli generated by an arbitrary waveform generator (RS components, RSDG2000X series). Neutral density filters (ThorLabs) in a motorized filter wheel were used to control intensity of light stimuli from 11.9 to 17.4 log photons $cm^2$/s. Devices were automatically controlled and synchronized by a Digidata 1440A digital I/O board (Axon Instruments, Molecular Devices) and a PC running WinWCO software (J Dempster, Strathclyde University, UK).

Responses were recorded to full-field chirp stimuli consisting of 3 s step from dark to 100% intensity, followed by 2 s of dark, 2 s at 50% intensity, 8 s temporal chirp (accelerating sinusoidal modulation at 100% contrast from 1 to 8 Hz at 1 Hz/s), 2 s at 50% intensity, 8 s contrast chirp (2 Hz sinusoidal modulation from 3% to 97% contrast), 2 s at 50% intensity and 3 s of dark. Chirp stimuli were presented from lowest to brightest intensity.

#### Quantification and statistical analysis

Unless otherwise specified, graphs show mean with error bars showing standard error of the mean, sample size is given in the figure legends and refers to number of retinal and LGN units. Comparisons between individual ReaChR Grm6 rd/rd and ReaChR Brn3c rd/rd units used Mann-Whitney U-tests, with significance determined as $p < 0.05$, at 15.95 log photons/$cm^2$/s for LGN and 16.97 log photons/$cm^2$/s for retina (unless stated otherwise). These irradiances were used as they produced responses closest to 75% maximum, providing a sub-saturating response with large sample size and good signal to noise ratio.

##### *Identifying light responsive units*

Peristimulus time histograms (PSTH) with 25 ms bin size were generated. Units with low spike firing rates (<10% of bins containing spiking activity) and spiking activity in <8 trials were excluded from further analysis. Light responsive (LR) units were identified using a shuffle test based on correlation across trials in response to entire chirp stimulus. A significance threshold of $p < 0.0001$ was used.

##### *Response amplitude and irradiance response curves*

Normalized firing rate was calculated by subtracting average baseline firing during 2 s before onset of 3 s step stimulus. Response amplitude was defined as maximum normalized firing rate during a 3 s step or 3 s after step stimulus to capture both ON and OFF responses. The response amplitude of units that were LR to brightest intensity tested (16.97 log photons/$cm^2$/s for retina and 17.4 log photons/$cm^2$/s for LGN) was then averaged for each retina or LGN electrode placement and plotted against stimulus intensity. For retina, these data were fit with irradiance response curve using Hill slope[42] with four free parameters (top, bottom, slope, and $EC_{50}$) to estimate photosensitivity.

##### *Quality index*

To assess response reproducibility, PSTH with 200 ms bin size was used to calculate to quality index,[40] and generated for LR units at brightest intensity tested for each genotype. This was calculated as:

$$\text{Quality Index} = \frac{\text{variance}\left[\text{mean}(\text{Spike Counts})_{\text{repeats}}\right]_{\text{time}}}{\text{mean}\left(\text{variance}[\text{Spike Counts}]_{\text{time}}\right)_{\text{repeats}}}$$

Spike counts are organized into response matrix of time bins × stimulus repeats, and mean, ( ), and variance, [ ], are calculated across the indicated dimension, either time "$)_{time}$," or repeats "$)_{repeats}$." The quality index scale is from 0 (entirely random activity between and across trials) to 1 (identical response across trials). To control for firing rate, which is closely related to response reproducibility,[81] we identified a sub-sample of Brn3c rd/rd and Grm6 rd/rd units with matched distributions for average firing rate across entire chirp stimulus. We then compared the quality index of these firing-rate-matched units.

##### *Response latency to step onset*

Latency to onset of step stimulus was based on PSTH with 1 ms bin size, smoothed with a 10 ms boxcar filter using MATLAB *filtfilt* function. Latency to step onset was calculated as timing of first bin to exceed 95% confidence limit in 300 ms after onset of step stimulus. This 95% confidence limit was based on 2 standard deviations of baseline firing during 300 ms before onset of light step. Units which did not exceed this threshold, such as OFF units, were excluded from this analysis.

***Response polarity and transience***

Units were grouped into response categories (ON transient, ON sustained, ON-OFF and OFF) using objective criteria, as described in Rodgers et al.[42] ON-OFF bias index[82] was used to assess response polarity and was calculated as ratio of spike firing during 500 ms after onset (ON firing) and 500 ms after offset (OFF firing) of light step. This produces a scale from −1 (firing to OFF only) to 0 (equal firing for ON and OFF) to 1 (firing to ON only).

Transience index[43,82] was used to test response persistence. PSTH with 25 ms bin size was normalized to maximum firing rate during 3 s steps. Area under the curve was then calculated for 1 s after stimulus onset for ON units (defined as ON-OFF bias index > −0.33) or 1 s after stimulus offset for OFF units (ON-OFF bias index < −0.33). This produces a scale from 0 (highly transient) to 1 (highly sustained with identical response across all bins tested).

***Analysis of ReaChR Brn3c rd/+ and WT retinas***

To identify Brn3c-positive units and compare their photoreceptor and ReaChR-driven activity, we identified units from ReaChR Brn3c rd/+ retinas that were both (1) LR at light intensity below threshold for ReaChR activation (13.95 log photons/cm$^2$/s) during perfusion with standard AMES and (2) LR at light intensity above threshold for ReaChR activation (15.95 log photons/cm$^2$/s) during perfusion with AMES containing synaptic blockers. Activity under former condition was defined as photoreceptor driven, while activity recorded under latter was defined as ReaChR driven.

For analysis of WT retinas across intensities, we compared units that were LR at intensities 13.95 and 15.95 log photons/cm$^2$/s during perfusion with standard AMES. As these mice do not possess ReaChR, responses are driven by rod and cone photoreceptors under both irradiance conditions.

***Contrast sensitivity***

To assess contrast sensitivity, we used PSTH with 25 ms bin size. Response amplitude to each sinusoidal modulation was calculated as maximum firing—minimum firing rate during 0.5 s each contrast was presented. These were then normalized to response amplitude during 0.5 s before contrast chirp onset and plotted against Michelson contrast and fit with the Naka-Rushton function[83] with four free parameters (top, bottom, slope, and $C_{50}$) using least-squares minimization. $C_{50}$ was constrained between 0 and 1, and slope was constrained between 0 and 10. Only units with curve fits where $R^2$ > 0.5 and spiking in >10% of bins were used for comparison of contrast sensitivity parameters.

***Temporal frequency tuning***

For temporal tuning, we used PSTH with 25 ms bin size. The mean response amplitude (based on maximum firing-minimum firing during each sinusoidal modulation) was calculated for each temporal frequency. These data were fitted with a half-Gaussian model[84] with five free parameters (low baseline, high baseline, Gaussian spread, peak response amplitude, and peak temporal frequency) using least-squares minimization. Peak temporal frequency was constrained between 1 and 8 Hz. Only units with curve fits where $R^2$ > 0.5, spiking in >10% of bins, and Gaussian spread >0.51 were used for comparison of temporal frequency tuning parameters.

***Community detection***

sPCs were generated for three parts of chirp stimulus (step at 0.5–4.5 s, temporal chirp at 6.5–15.5 s, and contrast chirp at 17.5–24.5 s) from PSTH with 50 ms bin size using the SPaSM toolbox.[85] sPCs were generated based on pooled data from WT, ReaChR Grm6 rd/rd, and ReaChR Brn3c rd/rd recordings. For retina data in Figure 7, data were used from LR units at 15.95 (ReaChR Grm6 and Brn3c) and 12.95 photons/cm$^2$/s (WT), while for LGN data were used from LR units at 16.97 (ReaChR Grm6 and Brn3c) and 14.99 photons/cm$^2$/s (WT). Units were randomly downsampled to match sample size of genotype with fewest units, $n$ = 702 units for retina and $n$ = 283 for LGN. After discarding sPCs accounting for <1% of variance, we extracted 57 sPCs for retina and 74 sPCs for dLGN with 5 non-zero time bins. sPCs were clustered using a Gaussian mixed model with random initialization. Optimal number of clusters was determined by lowest Bayesian information criteria and Bayes factor <6 (as in Caval-Holme et al.[86]). Clustering was repeated 50 times and used to generate a pairwise similarity matrix. A community detection algorithm based on this similarity matrix, using the Brain connectivity toolbox,[87] was then used to group units into communities. Communities with <5 units were excluded from further analysis. Distribution of units across communities was compared between genotypes using a shuffle test, as described previously.[42]

## DATA AND CODE AVAILABILITY

Data reported in this paper will be shared by the lead contact upon request. Any additional information required to reanalyze the data reported in this paper is available from the lead contact upon request. The *ReaChR Brn3c rd* and *ReaChR Grm6 rd* transgenic mice are available subject to the completion of material transfer agreements.

## ACKNOWLEDGMENTS

This work was funded by MRC grant (MR/S026266/1) awarded to M.W.H., S.H., S.N.P., and R.J.L. M.L. was funded by grants from the Pro Retina Foundation (Pro-Re/Projekt/Gi-Wh-Li.04.2021) and the German Research Foundation (LI 2846/6–1). A.E.A. is funded by a Sir Henry Dale Fellowship, jointly funded by the Wellcome Trust and the Royal Society (grant no. 218556/Z/19/Z). R.S. is funded by a Sir Henry Dale Fellowship, jointly funded by the Wellcome Trust and the Royal Society (grant no. 220163/Z/20/Z). J.R. is funded by a Fight for Sight Small Grant Award (RESSGA2303). T.C.B. is funded by Unitatea Executiva pentru Finantarea Invatamantului Superior, a Cercetarii, si Inovarii (grant no. PNIII-P4-PCE-2021-0333).

## AUTHOR CONTRIBUTIONS

J.R., S.H., M.W.H., and R.J.L. designed the study. S.H. and J.R. performed the experiments. T.C.B. provided Brn3c Cre mice. M.L., A.S.E., A.E.A., S.N.P., and R.S. provided tools for data analysis, which was conducted by J.R. J.R., M.W.H., and R.J.L. wrote the manuscript, which was edited and approved by all authors.

## DECLARATION OF INTERESTS

R.J.L. and J.R. are named inventors on patent applications for the use of animal opsins in optogenetics. R.J.L. has received investigator-initiated research funding from Kubota Vision Inc. and acted as a consultant for Kubota Vision Inc.

## SUPPLEMENTAL INFORMATION

Supplemental information can be found online at https://doi.org/10.1016/j.ymthe.2025.01.030.

## REFERENCES


1. Baker, C.K., and Flannery, J.G. (2018). Innovative Optogenetic Strategies for Vision Restoration. Front. Cell. Neurosci. *12*, 316. https://doi.org/10.3389/fncel.2018.00316.
2. Lindner, M., Gilhooley, M.J., Hughes, S., and Hankins, M.W. (2022). Optogenetics for visual restoration: From proof of principle to translational challenges. Prog. Retin. Eye Res. *91*, 101089. https://doi.org/10.1016/j.preteyeres.2022.101089.
3. Parnami, K., and Bhattacharyya, A. (2023). Current approaches to vision restoration using optogenetic therapy. Front. Cell. Neurosci. *17*, 1236826. https://doi.org/10.3389/fncel.2023.1236826.
4. Sahel, J.-A., Boulanger-Scemama, E., Pagot, C., Arleo, A., Galluppi, F., Martel, J.N., Esposti, S.D., Delaux, A., de Saint Aubert, J.-B., de Montleau, C., et al. (2021). Partial recovery of visual function in a blind patient after optogenetic therapy. Nat. Med. *27*, 1223–1229. https://doi.org/10.1038/s41591-021-01351-4.
5. Varin, J., Bouzidi, N., Gauvain, G., Joffrois, C., Desrosiers, M., Robert, C., De Sousa Dias, M.M., Neuillé, M., Michiels, C., Nassisi, M., et al. (2021). Substantial restoration of night vision in adult mice with congenital stationary night blindness. Mol. Ther. Methods Clin. Dev. *22*, 15–25. https://doi.org/10.1016/j.omtm.2021.05.008.
6. Miyadera, K., Santana, E., Roszak, K., Iffrig, S., Visel, M., Iwabe, S., Boyd, R.F., Bartoe, J.T., Sato, Y., Gray, A., et al. (2022). Targeting ON-bipolar cells by AAV gene therapy stably reverses *LRIT3* -congenital stationary night blindness. Proc. Natl. Acad. Sci. USA *119*, e2117038119. https://doi.org/10.1073/pnas.2117038119.
7. Fischer, M.D., Michalakis, S., Wilhelm, B., Zobor, D., Muehlfriedel, R., Kohl, S., Weisschuh, N., Ochakovski, G.A., Klein, R., Schoen, C., et al. (2020). Safety and Vision Outcomes of Subretinal Gene Therapy Targeting Cone Photoreceptors in Achromatopsia. JAMA Ophthalmol. *138*, 1–9. https://doi.org/10.1001/jamaophthalmol.2020.1032.
8. Komáromy, A.M., Alexander, J.J., Rowlan, J.S., Garcia, M.M., Chiodo, V.A., Kaya, A., Tanaka, J.C., Acland, G.M., Hauswirth, W.W., and Aguirre, G.D. (2010). Gene therapy rescues cone function in congenital achromatopsia. Hum. Mol. Genet. *19*, 2581–2593. https://doi.org/10.1093/hmg/ddq136.
9. Khabou, H., Orendorff, E., Trapani, F., Rucli, M., Desrosiers, M., Yger, P., Dalkara, D., and Marre, O. (2023). Optogenetic targeting of AII amacrine cells restores retinal computations performed by the inner retina. Mol. Ther. Methods Clin. Dev. *31*, 101107. https://doi.org/10.1016/j.omtm.2023.09.003.
10. Busskamp, V., Duebel, J., Balya, D., Fradot, M., Viney, T.J., Siegert, S., Groner, A.C., Cabuy, E., Forster, V., Seeliger, M., et al. (2010). Genetic Reactivation of Cone Photoreceptors Restores Visual Responses in Retinitis Pigmentosa. Science *329*, 413–417. https://doi.org/10.1126/science.1190897.
11. Cehajic-Kapetanovic, J., Eleftheriou, C., Allen, A.E., Milosavljevic, N., Pienaar, A., Bedford, R., Davis, K.E., Bishop, P.N., and Lucas, R.J. (2015). Restoration of Vision with Ectopic Expression of Human Rod Opsin. Curr. Biol. *25*, 2111–2122. https://doi.org/10.1016/j.cub.2015.07.029.
12. Cronin, T., Vandenberghe, L.H., Hantz, P., Juttner, J., Reimann, A., Kacsó, A.E., Huckfeldt, R.M., Busskamp, V., Kohler, H., Lagali, P.S., et al. (2014). Efficient transduction and optogenetic stimulation of retinal bipolar cells by a synthetic adeno-associated virus capsid and promoter. EMBO Mol. Med. *6*, 1175–1190. https://doi.org/10.15252/emmm.201404077.
13. Doroudchi, M.M., Greenberg, K.P., Liu, J., Silka, K.A., Boyden, E.S., Lockridge, J.A., Arman, A.C., Janani, R., Boye, S.E., Boye, S.L., et al. (2011). Virally delivered Channelrhodopsin-2 Safely and Effectively Restores Visual Function in Multiple Mouse Models of Blindness. Mol. Ther. *19*, 1220–1229. https://doi.org/10.1038/mt.2011.69.
14. Gaub, B.M., Berry, M.H., Holt, A.E., Isacoff, E.Y., and Flannery, J.G. (2015). Optogenetic Vision Restoration Using Rhodopsin for Enhanced Sensitivity. Mol. Ther. *23*, 1562–1571. https://doi.org/10.1038/mt.2015.121.
15. Kralik, J., van Wyk, M., Stocker, N., and Kleinlogel, S. (2022). Bipolar cell targeted optogenetic gene therapy restores parallel retinal signaling and high-level vision in the degenerated retina. Commun. Biol. *5*, 1116. https://doi.org/10.1038/s42003-022-04016-1.
16. Lagali, P.S., Balya, D., Awatramani, G.B., Münch, T.A., Kim, D.S., Busskamp, V., Cepko, C.L., and Roska, B. (2008). Light-activated channels targeted to ON bipolar cells restore visual function in retinal degeneration. Nat. Neurosci. *11*, 667–675. https://doi.org/10.1038/nn.2117.
17. Macé, E., Caplette, R., Marre, O., Sengupta, A., Chaffiol, A., Barbe, P., Desrosiers, M., Bamberg, E., Sahel, J.-A., Picaud, S., et al. (2015). Targeting Channelrhodopsin-2 to ON-bipolar Cells With Vitreally Administered AAV Restores ON and OFF Visual Responses in Blind Mice. Mol. Ther. *23*, 7–16. https://doi.org/10.1038/mt.2014.154.
18. Berry, M.H., Holt, A., Salari, A., Veit, J., Visel, M., Levitz, J., Aghi, K., Gaub, B.M., Sivyer, B., Flannery, J.G., and Isacoff, E.Y. (2019). Restoration of high-sensitivity and adapting vision with a cone opsin. Nat. Commun. *10*, 1221. https://doi.org/10.1038/s41467-019-09124-x.
19. Bi, A., Cui, J., Ma, Y.-P., Olshevskaya, E., Pu, M., Dizhoor, A.M., and Pan, Z.-H. (2006). Ectopic Expression of a Microbial-Type Rhodopsin Restores Visual Responses in Mice with Photoreceptor Degeneration. Neuron *50*, 23–33. https://doi.org/10.1016/j.neuron.2006.02.026.
20. De Silva, S.R., Barnard, A.R., Hughes, S., Tam, S.K.E., Martin, C., Singh, M.S., Barnea-Cramer, A.O., McClements, M.E., During, M.J., Peirson, S.N., et al. (2017). Long-term restoration of visual function in end-stage retinal degeneration using subretinal human melanopsin gene therapy. Proc. Natl. Acad. Sci. USA *114*, 11211–11216. https://doi.org/10.1073/pnas.1701589114.
21. Ferrari, U., Deny, S., Sengupta, A., Caplette, R., Trapani, F., Sahel, J.-A., Dalkara, D., Picaud, S., Duebel, J., and Marre, O. (2020). Towards optogenetic vision restoration with high resolution. PLoS Comput. Biol. *16*, e1007857. https://doi.org/10.1371/journal.pcbi.1007857.
22. Gauvain, G., Akolkar, H., Chaffiol, A., Arcizet, F., Khoei, M.A., Desrosiers, M., Jaillard, C., Caplette, R., Marre, O., Bertin, S., et al. (2021). Optogenetic therapy: high spatiotemporal resolution and pattern discrimination compatible with vision restoration in non-human primates. Commun. Biol. *4*, 125. https://doi.org/10.1038/s42003-020-01594-w.
23. Lin, B., Koizumi, A., Tanaka, N., Panda, S., and Masland, R.H. (2008). Restoration of visual function in retinal degeneration mice by ectopic expression of melanopsin. Proc. Natl. Acad. Sci. USA *105*, 16009–16014. https://doi.org/10.1073/pnas.0806114105.
24. Sengupta, A., Chaffiol, A., Macé, E., Caplette, R., Desrosiers, M., Lampič, M., Forster, V., Marre, O., Lin, J.Y., Sahel, J.-A., et al. (2016). Red-shifted channelrhodopsin stimulation restores light responses in blind mice, macaque retina, and human retina. EMBO Mol. Med. *8*, 1248–1264. https://doi.org/10.15252/emmm.201505699.
25. Thyagarajan, S., van Wyk, M., Lehmann, K., Löwel, S., Feng, G., and Wässle, H. (2010). Visual Function in Mice with Photoreceptor Degeneration and Transgenic Expression of Channelrhodopsin 2 in Ganglion Cells. J. Neurosci. *30*, 8745–8758. https://doi.org/10.1523/JNEUROSCI.4417-09.2010.
26. Nieuwenhuis, B., Laperrousaz, E., Tribble, J.R., Verhaagen, J., Fawcett, J.W., Martin, K.R., Williams, P.A., and Osborne, A. (2023). Improving adeno-associated viral (AAV) vector-mediated transgene expression in retinal ganglion cells: comparison of five promoters. Gene Ther. *30*, 503–519. https://doi.org/10.1038/s41434-022-00380-z.
27. Gilhooley, M.J., Lindner, M., Palumaa, T., Hughes, S., Peirson, S.N., and Hankins, M.W. (2022). A systematic comparison of optogenetic approaches to visual restoration. Mol. Ther. Methods Clin. Dev. *25*, 111–123. https://doi.org/10.1016/j.omtm.2022.03.003.
28. Hulliger, E.C., Hostettler, S.M., and Kleinlogel, S. (2020). Empowering Retinal Gene Therapy with a Specific Promoter for Human Rod and Cone ON-Bipolar Cells. Mol. Ther. Methods Clin. Dev. *17*, 505–519. https://doi.org/10.1016/j.omtm.2020.03.003.
29. Lu, Q., Ganjawala, T.H., Krstevski, A., Abrams, G.W., and Pan, Z.-H. (2020). Comparison of AAV-Mediated Optogenetic Vision Restoration between Retinal Ganglion Cell Expression and ON Bipolar Cell Targeting. Mol. Ther. Methods Clin. Dev. *18*, 15–23. https://doi.org/10.1016/j.omtm.2020.05.009.
30. Lu, Q., Ganjawala, T.H., Ivanova, E., Cheng, J.G., Troilo, D., and Pan, Z.-H. (2016). AAV-mediated transduction and targeting of retinal bipolar cells with improved mGluR6 promoters in rodents and primates. Gene Ther. *23*, 680–689. https://doi.org/10.1038/gt.2016.42.

31. Scalabrino, M.L., Boye, S.L., Fransen, K.M.H., Noel, J.M., Dyka, F.M., Min, S.H., Ruan, Q., De Leeuw, C.N., Simpson, E.M., Gregg, R.G., et al. (2015). Intravitreal delivery of a novel AAV vector targets ON bipolar cells and restores visual function in a mouse model of complete congenital stationary night blindness. Hum. Mol. Genet. *24*, 6229–6239. https://doi.org/10.1093/hmg/ddv341.

32. McClements, M.E., Staurenghi, F., Visel, M., Flannery, J.G., MacLaren, R.E., and Cehajic-Kapetanovic, J. (2021). AAV Induced Expression of Human Rod and Cone Opsin in Bipolar Cells of a Mouse Model of Retinal Degeneration. Biomed. Res. Int. *2021*, 1–8. https://doi.org/10.1155/2021/4014797.

33. Korecki, A.J., Cueva-Vargas, J.L., Fornes, O., Agostinone, J., Farkas, R.A., Hickmott, J.W., Lam, S.L., Mathelier, A., Zhou, M., Wasserman, W.W., et al. (2021). Human MiniPromoters for ocular-rAAV expression in ON bipolar, cone, corneal, endothelial, Müller glial, and PAX6 cells. Gene Ther. *28*, 351–372. https://doi.org/10.1038/s41434-021-00227-z.

34. Dalkara, D., Byrne, L.C., Klimczak, R.R., Visel, M., Yin, L., Merigan, W.H., Flannery, J.G., and Schaffer, D.V. (2013). In Vivo–Directed Evolution of a New Adeno-Associated Virus for Therapeutic Outer Retinal Gene Delivery from the Vitreous. Sci. Transl. Med. *5*, 189ra76. https://doi.org/10.1126/scitranslmed.3005708.

35. Hickey, D.G., Edwards, T.L., Barnard, A.R., Singh, M.S., de Silva, S.R., McClements, M.E., Flannery, J.G., Hankins, M.W., and MacLaren, R.E. (2017). Tropism of engineered and evolved recombinant AAV serotypes in the rd1 mouse and ex vivo primate retina. Gene Ther. *24*, 787–800. https://doi.org/10.1038/gt.2017.85.

36. Petrs-Silva, H., Dinculescu, A., Li, Q., Deng, W.-T., Pang, J.J., Min, S.-H., Chiodo, V., Neeley, A.W., Govindasamy, L., Bennett, A., et al. (2011). Novel Properties of Tyrosine-mutant AAV2 Vectors in the Mouse Retina. Mol. Ther. *19*, 293–301. https://doi.org/10.1038/mt.2010.234.

37. Jones, B.W., Pfeiffer, R.L., Ferrell, W.D., Watt, C.B., Marmor, M., and Marc, R.E. (2016). Retinal remodeling in human retinitis pigmentosa. Exp. Eye Res. *150*, 149–165. https://doi.org/10.1016/j.exer.2016.03.018.

38. Pfeiffer, R.L., Marc, R.E., and Jones, B.W. (2020). Persistent remodeling and neurodegeneration in late-stage retinal degeneration. Prog. Retin. Eye Res. *74*, 100771. https://doi.org/10.1016/j.preteyeres.2019.07.004.

39. Puthussery, T., Gayet-Primo, J., Pandey, S., Duvoisin, R.M., and Taylor, W.R. (2009). Differential loss and preservation of glutamate receptor function in bipolar cells in the rd10 mouse model of retinitis pigmentosa. Eur. J. Neurosci. *29*, 1533–1542. https://doi.org/10.1111/j.1460-9568.2009.06728.x.

40. Baden, T., Berens, P., Franke, K., Román Rosón, M., Bethge, M., and Euler, T. (2016). The functional diversity of retinal ganglion cells in the mouse. Nature *529*, 345–350. https://doi.org/10.1038/nature16468.

41. Goetz, J., Jessen, Z.F., Jacobi, A., Mani, A., Cooler, S., Greer, D., Kadri, S., Segal, J., Shekhar, K., Sanes, J.R., and Schwartz, G.W. (2022). Unified classification of mouse retinal ganglion cells using function, morphology, and gene expression. Cell Rep. *40*, 111040. https://doi.org/10.1016/j.celrep.2022.111040.

42. Rodgers, J., Hughes, S., Lindner, M., Allen, A.E., Ebrahimi, A.S., Storchi, R., Peirson, S.N., Lucas, R.J., and Hankins, M.W. (2023). Functional integrity of visual coding following advanced photoreceptor degeneration. Curr. Biol. *33*, 474–486.e5. https://doi.org/10.1016/j.cub.2022.12.026.

43. Lindner, M., Gilhooley, M.J., Peirson, S.N., Hughes, S., and Hankins, M.W. (2021). The functional characteristics of optogenetic gene therapy for vision restoration. Cell. Mol. Life Sci. *78*, 1597–1613. https://doi.org/10.1007/s00018-020-03597-6.

44. Chang, B., Hawes, N.L., Hurd, R.E., Davisson, M.T., Nusinowitz, S., and Heckenlively, J.R. (2002). Retinal degeneration mutants in the mouse. Vis. Res. *42*, 517–525. https://doi.org/10.1016/S0042-6989(01)00146-8.

45. Pittler, S.J., and Baehr, W. (1991). Identification of a nonsense mutation in the rod photoreceptor cGMP phosphodiesterase beta-subunit gene of the rd mouse. Proc. Natl. Acad. Sci. USA *88*, 8322–8326. https://doi.org/10.1073/pnas.88.19.8322.

46. Hooks, B.M., Lin, J.Y., Guo, C., and Svoboda, K. (2015). Dual-channel circuit mapping reveals sensorimotor convergence in the primary motor cortex. J. Neurosci. *35*, 4418–4426. https://doi.org/10.1523/JNEUROSCI.3741-14.2015.

47. Morgans, C.W., Zhang, J., Jeffrey, B.G., Nelson, S.M., Burke, N.S., Duvoisin, R.M., and Brown, R.L. (2009). TRPM1 is required for the depolarizing light response in retinal ON-bipolar cells. Proc. Natl. Acad. Sci. USA *106*, 19174–19178. https://doi.org/10.1073/pnas.0908711106.

48. Parmhans, N., Fuller, A.D., Nguyen, E., Chuang, K., Swygart, D., Wienbar, S.R., Lin, T., Kozmik, Z., Dong, L., Schwartz, G.W., and Badea, T.C. (2021). Identification of retinal ganglion cell types and brain nuclei expressing the transcription factor Brn3c/Pou4f3 using a Cre recombinase knock-in allele. J. Comp. Neurol. *529*, 1926–1953. https://doi.org/10.1002/cne.25065.

49. Lin, J.Y., Knutsen, P.M., Muller, A., Kleinfeld, D., and Tsien, R.Y. (2013). ReaChR: a red-shifted variant of channelrhodopsin enables deep transcranial optogenetic excitation. Nat. Neurosci. *16*, 1499–1508. https://doi.org/10.1038/nn.3502.

50. Procyk, C.A., Allen, A.E., Martial, F.P., and Lucas, R.J. (2019). Visual responses in the dorsal lateral geniculate nucleus at early stages of retinal degeneration in $rd^{1}$ PDE6β mice. J. Neurophysiol. *122*, 1753–1764. https://doi.org/10.1152/jn.00231.2019.

51. Tikidji-Hamburyan, A., Reinhard, K., Seitter, H., Hovhannisyan, A., Procyk, C.A., Allen, A.E., Schenk, M., Lucas, R.J., and Münch, T.A. (2015). Retinal output changes qualitatively with every change in ambient illuminance. Nat. Neurosci. *18*, 66–74. https://doi.org/10.1038/nn.3891.

52. Bloomfield, S.A., and Dacheux, R.F. (2001). Rod Vision: Pathways and Processing in the Mammalian Retina. Prog. Retin. Eye Res. *20*, 351–384. https://doi.org/10.1016/S1350-9462(00)00031-8.

53. Famiglietti, E.V., and Kolb, H. (1975). A bistratified amacrine cell and synaptic circuitry in the inner plexiform layer of the retina. Brain Res. *84*, 293–300. https://doi.org/10.1016/0006-8993(75)90983-X.

54. Field, G.D., Sampath, A.P., and Rieke, F. (2005). RETINAL PROCESSING NEAR ABSOLUTE THRESHOLD: From Behavior to Mechanism. Annu. Rev. Physiol. *67*, 491–514. https://doi.org/10.1146/annurev.physiol.67.031103.151256.

55. Graydon, C.W., Lieberman, E.E., Rho, N., Briggman, K.L., Singer, J.H., and Diamond, J.S. (2018). Synaptic Transfer between Rod and Cone Pathways Mediated by AII Amacrine Cells in the Mouse Retina. Curr. Biol. *28*, 2739–2751.e3. https://doi.org/10.1016/j.cub.2018.06.063.

56. Grimes, W.N., Songco-Aguas, A., and Rieke, F. (2018). Parallel Processing of Rod and Cone Signals: Retinal Function and Human Perception. Annu. Rev. Vis. Sci. *4*, 123–141. https://doi.org/10.1146/annurev-vision-091517-034055.

57. Strettoi, E., Raviola, E., and Dacheux, R.F. (1992). Synaptic connections of the narrow-field, bistratified rod amacrine cell (AII) in the rabbit retina. J. Comp. Neurol. *325*, 152–168. https://doi.org/10.1002/cne.903250203.

58. Tsukamoto, Y., and Omi, N. (2017). Classification of Mouse Retinal Bipolar Cells: Type-Specific Connectivity with Special Reference to Rod-Driven AII Amacrine Pathways. Front. Neuroanat. *11*, 92. https://doi.org/10.3389/fnana.2017.00092.

59. Ichinose, T., Fyk-Kolodziej, B., and Cohn, J. (2014). Roles of ON Cone Bipolar Cell Subtypes in Temporal Coding in the Mouse Retina. J. Neurosci. *34*, 8761–8771. https://doi.org/10.1523/JNEUROSCI.3965-13.2014.

60. Baden, T., Nikolaev, A., Esposti, F., Dreosti, E., Odermatt, B., and Lagnado, L. (2014). A Synaptic Mechanism for Temporal Filtering of Visual Signals. PLoS Biol. *12*, e1001972. https://doi.org/10.1371/journal.pbio.1001972.

61. Molnar, A., and Werblin, F. (2007). Inhibitory Feedback Shapes Bipolar Cell Responses in the Rabbit Retina. J. Neurophysiol. *98*, 3423–3435. https://doi.org/10.1152/jn.00838.2007.

62. Murphy-Baum, B.L., and Taylor, W.R. (2018). Diverse inhibitory and excitatory mechanisms shape temporal tuning in transient OFF α ganglion cells in the rabbit retina. J. Physiol. *596*, 477–495. https://doi.org/10.1113/JP275195.

63. Ganczer, A., Szarka, G., Balogh, M., Hoffmann, G., Tengölics, Á.J., Kenyon, G., Kovács-Öller, T., and Völgyi, B. (2022). Transience of the Retinal Output Is Determined by a Great Variety of Circuit Elements. Cells *11*, 810. https://doi.org/10.3390/cells11050810.

64. Goldin, M.A., Lefebvre, B., Virgili, S., Pham Van Cang, M.K., Ecker, A., Mora, T., Ferrari, U., and Marre, O. (2022). Context-dependent selectivity to natural images in the retina. Nat. Commun. *13*, 5556. https://doi.org/10.1038/s41467-022-33242-8.

65. Pearson, J.T., and Kerschensteiner, D. (2015). Ambient illumination switches contrast preference of specific retinal processing streams. J. Neurophysiol. *114*, 540–550. https://doi.org/10.1152/jn.00360.2015.

66. Gilhooley, M.J., Hickey, D.G., Lindner, M., Palumaa, T., Hughes, S., Peirson, S.N., MacLaren, R.E., and Hankins, M.W. (2021). ON-bipolar cell gene expression during

retinal degeneration: Implications for optogenetic visual restoration. Exp. Eye Res. *207*, 108553. https://doi.org/10.1016/j.exer.2021.108553.

67. Dryja, T.P., McGee, T.L., Berson, E.L., Fishman, G.A., Sandberg, M.A., Alexander, K.R., Derlacki, D.J., and Rajagopalan, A.S. (2005). Night blindness and abnormal cone electroretinogram ON responses in patients with mutations in the GRM6 gene encoding mGluR6. Proc. Natl. Acad. Sci. USA *102*, 4884–4889. https://doi.org/10.1073/pnas.0501233102.

68. Peachey, N.S., Ray, T.A., Florijn, R., Rowe, L.B., Sjoerdsma, T., Contreras-Alcantara, S., Baba, K., Tosini, G., Pozdeyev, N., Iuvone, P.M., et al. (2012). GPR179 is required for depolarizing bipolar cell function and is mutated in autosomal-recessive complete congenital stationary night blindness. Am. J. Hum. Genet. *90*, 331–339. https://doi.org/10.1016/j.ajhg.2011.12.006.

69. Li, Z., Sergouniotis, P.I., Michaelides, M., Mackay, D.S., Wright, G.A., Devery, S., Moore, A.T., Holder, G.E., Robson, A.G., and Webster, A.R. (2009). Recessive Mutations of the Gene TRPM1 Abrogate ON Bipolar Cell Function and Cause Complete Congenital Stationary Night Blindness in Humans. Am. J. Hum. Genet. *85*, 711–719. https://doi.org/10.1016/j.ajhg.2009.10.003.

70. Chang, L., Ran, Y., Yang, M., Auferkorte, O., Butz, E., Hüser, L., Haverkamp, S., Euler, T., and Schubert, T. (2023). Spike desensitisation as a mechanism for high-contrast selectivity in retinal ganglion cells. Front. Cell. Neurosci. *17*, 1337768. https://doi.org/10.3389/fncel.2023.1337768.

71. Nirenberg, S., and Pandarinath, C. (2012). Retinal prosthetic strategy with the capacity to restore normal vision. Proc. Natl. Acad. Sci. USA *109*, 15012–15017. https://doi.org/10.1073/pnas.1207035109.

72. Demb, J.B., and Singer, J.H. (2015). Functional Circuitry of the Retina. Annu. Rev. Vis. Sci. *1*, 263–289. https://doi.org/10.1146/annurev-vision-082114-035334.

73. Dunn, F.A., Lankheet, M.J., and Rieke, F. (2007). Light adaptation in cone vision involves switching between receptor and post-receptor sites. Nature *449*, 603–606. https://doi.org/10.1038/nature06150.

74. Ke, J.-B., Wang, Y.V., Borghuis, B.G., Cembrowski, M.S., Riecke, H., Kath, W.L., Demb, J.B., and Singer, J.H. (2014). Adaptation to background light enables contrast coding at rod bipolar cell synapses. Neuron *81*, 388–401. https://doi.org/10.1016/j.neuron.2013.10.054.

75. Rieke, F., and Rudd, M.E. (2009). The Challenges Natural Images Pose for Visual Adaptation. Neuron *64*, 605–616. https://doi.org/10.1016/j.neuron.2009.11.028.

76. Shapley, R., and Enroth-Cugell, C. (1984). Chapter 9 Visual adaptation and retinal gain controls. Prog. Retin. Res. *3*, 263–346. https://doi.org/10.1016/0278-4327(84)90011-7.

77. Dalkara, D., Kolstad, K.D., Caporale, N., Visel, M., Klimczak, R.R., Schaffer, D.V., and Flannery, J.G. (2009). Inner Limiting Membrane Barriers to AAV-mediated Retinal Transduction From the Vitreous. Mol. Ther. *17*, 2096–2102. https://doi.org/10.1038/mt.2009.181.

78. Bae, J.A., Mu, S., Kim, J.S., Turner, N.L., Tartavull, I., Kemnitz, N., Jordan, C.S., Norton, A.D., Silversmith, W.M., Prentki, R., et al. (2018). Digital Museum of Retinal Ganglion Cells with Dense Anatomy and Physiology. Cell *173*, 1293–1306.e19. https://doi.org/10.1016/j.cell.2018.04.040.

79. Hughes, S., Watson, T.S., Foster, R.G., Peirson, S.N., and Hankins, M.W. (2013). Nonuniform Distribution and Spectral Tuning of Photosensitive Retinal Ganglion Cells of the Mouse Retina. Curr. Biol. *23*, 1696–1701. https://doi.org/10.1016/j.cub.2013.07.010.

80. Pachitariu, M., Sridhar, S., Pennington, J., and Stringer, C. (2024). Spike sorting with Kilosort4. Nat. Methods *21*, 914–921. https://doi.org/10.1038/s41592-024-02232-7.

81. Milosavljevic, N., Storchi, R., Eleftheriou, C.G., Colins, A., Petersen, R.S., and Lucas, R.J. (2018). Photoreceptive retinal ganglion cells control the information rate of the optic nerve. Proc. Natl. Acad. Sci. USA *115*, E11817–E11826. https://doi.org/10.1073/pnas.1810701115.

82. Farrow, K., and Masland, R.H. (2011). Physiological clustering of visual channels in the mouse retina. J. Neurophysiol. *105*, 1516–1530. https://doi.org/10.1152/jn.00331.2010.

83. Albrecht, D.G., and Hamilton, D.B. (1982). Striate cortex of monkey and cat: contrast response function. J. Neurophysiol. *48*, 217–237. https://doi.org/10.1152/jn.1982.48.1.217.

84. Grubb, M.S., and Thompson, I.D. (2003). Quantitative characterization of visual response properties in the mouse dorsal lateral geniculate nucleus. J. Neurophysiol. *90*, 3594–3607. https://doi.org/10.1152/jn.00699.2003.

85. Sjöstrand, K., Clemmensen, L.H., Larsen, R., Einarsson, G., and Ersbøll, B. (2018). SpaSM: A MATLAB Toolbox for Sparse Statistical Modeling. J. Stat. Softw. *84*, 1–37. https://doi.org/10.18637/jss.v084.i10.

86. Caval-Holme, F., Zhang, Y., and Feller, M.B. (2019). Gap Junction Coupling Shapes the Encoding of Light in the Developing Retina. Curr. Biol. *29*, 4024–4035.e5. https://doi.org/10.1016/j.cub.2019.10.025.

87. Rubinov, M., and Sporns, O. (2010). Complex network measures of brain connectivity: Uses and interpretations. NeuroImage *52*, 1059–1069. https://doi.org/10.1016/j.neuroimage.2009.10.003.

YMTHE, Volume *33*

# Supplemental Information

# Enhanced restoration of visual code after targeting ON bipolar cells compared with retinal ganglion cells with optogenetic therapy

**Jessica Rodgers, Steven Hughes, Aghileh S. Ebrahimi, Annette E. Allen, Riccardo Storchi, Moritz Lindner, Stuart N. Peirson, Tudor C. Badea, Mark W. Hankins, and Robert J. Lucas**

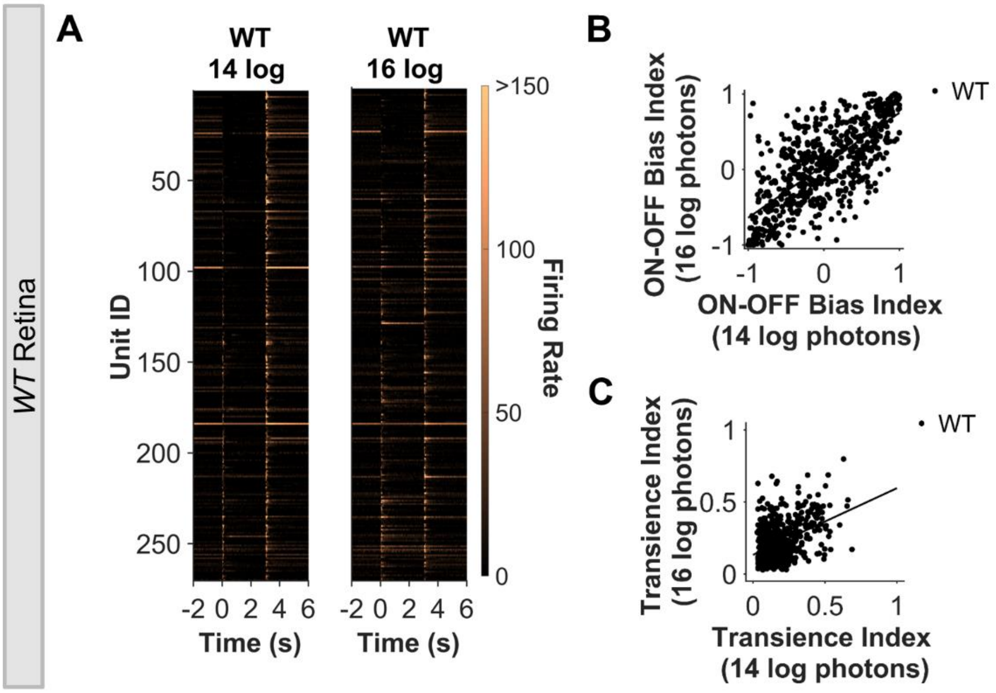


**Figure S1**

**A)** Heat map of mean PSTH for step stimulus (ON from 0-3s) for LR units in WT retina ordered by bias index from OFF (top) to ON (high) under 13.95 and 15.95 log photons/cm$^2$/s. Each row represents an individual unit. In the left and right heatmaps, each row represents the same unit recorded under different conditions (ranked based on ON-OFF bias for 13.95 log photons).

**B)** Scatterplot of ON-OFF bias index for same WT units at 13.95 and 15.95 log photons/cm$^2$/s.

**C)** Scatterplot of Transience index for WT units at 13.95 and 15.95 log photons/cm$^2$/s.

Data are from N = 630 WT retinal units (must be LR under both intensities to be included)

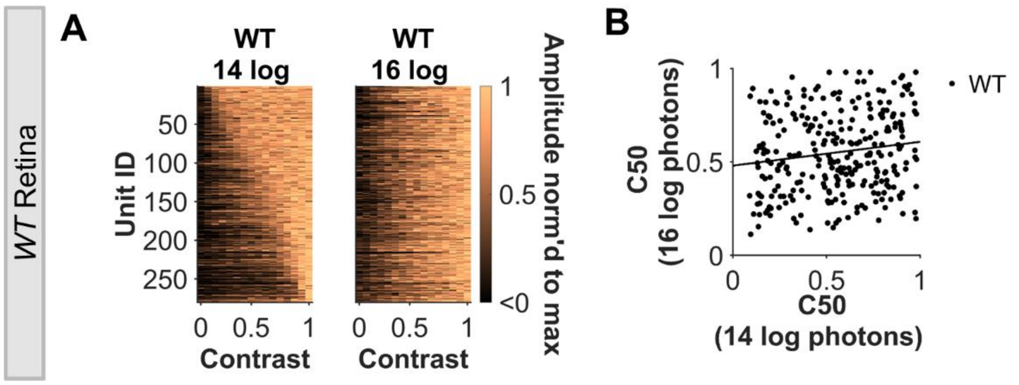


**Figure S2**

**A)** Heat map of maximum normalised response amplitude across contrasts for LR WT retinal units. Each row represents an individual unit. In the left and right heatmaps, each row represents the same unit recorded under different conditions (ranked by C50 for 13.95 log).

**B)** Scatterplot of C50 for same WT units at 13.95 and 15.95 log photons/cm$^2$/s.

Data are from N = 280 WT retinal units.

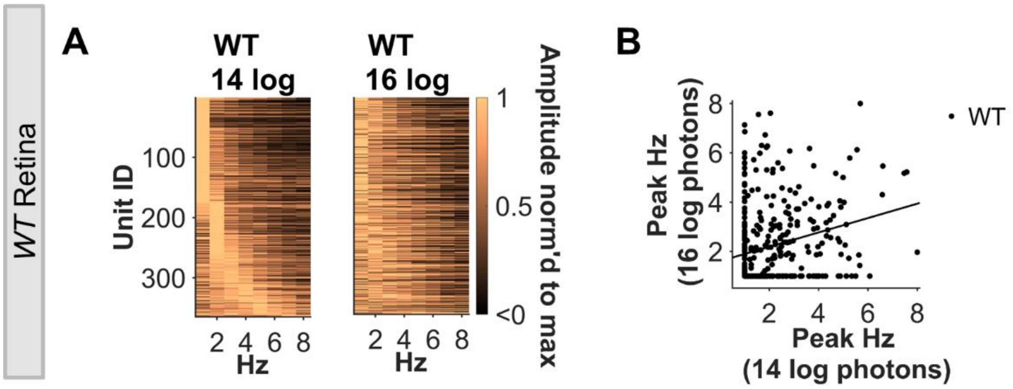


**Figure S3**

**A)** Heat map of maximum normalised response amplitude across frequencies for LR WT retinal units. Each row represents an individual unit. In left and right heatmaps, each row represents the same unit recorded under different conditions (ranked by peak TF for 13.95 log photons). **B)** Scatterplot of peak temporal frequency (peak Hz) for same WT units at 13.95 and 15.95 log photons/cm$^2$/s.

Data are from N = 369 WT retinal units.

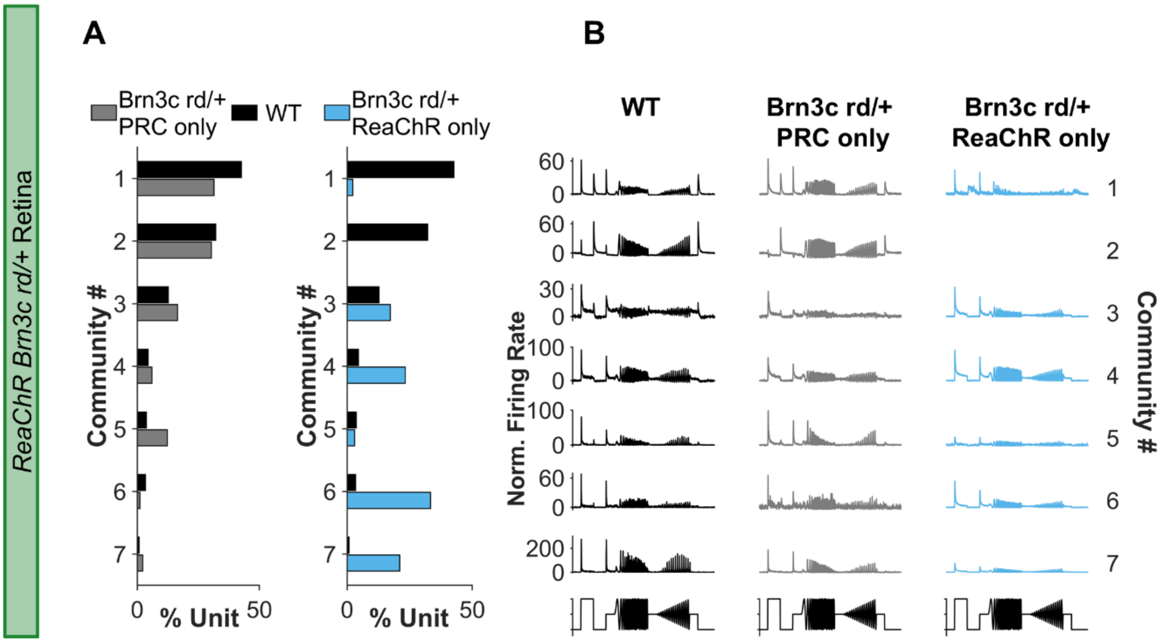


**Figure S4**

Community detection used data from LR retinal units at 13.95 photons/cm$^2$/s (WT and Brn3c rd/+ PRC only) and 15.95 photons/cm$^2$/s (Brn3c rd/+ ReaChR only). Units were randomly down-sampled to match sample size of genotype with fewest units, n = 267 units for each group. After discarding sPCs accounting for <1% of variance, we extracted 52 sPCs. Data for Brn3c rd/+ PRC and ReaChR only groups is within-subjects (ie: units are identical for both conditions), recorded under different conditions (see Figure 4b for further details). Data from each genotype is shown for communities with sufficient units to calculate mean (N ≥ 3).

**A)** Distribution of units across communities.

**B)** Mean baseline-subtracted firing rate for units from each community
